# Disrupted presynaptic nectin1-based neuronal adhesion in the entorhinal-hippocampal circuit contributes to early-life stress-induced memory deficits

**DOI:** 10.1038/s41398-022-01908-y

**Published:** 2022-04-04

**Authors:** Chen Wu, Qian Gong, Xue Xu, Ping Fang, Chi Wang, Jing-Ying Yu, Xing-Xing Wang, San-Hua Fang, Wen-Juan Chen, Hui-Fang Lou, Yu-Hui Liu, Liang Wang, Yi-Jun Liu, Wei Chen, Xiao-Dong Wang

**Affiliations:** 1 Department of Neurobiology and Department of Psychiatry of Sir Run Run Shaw Hospital, Zhejiang University School of Medicine, 310058 Hangzhou, China; 2 NHC and CAMS Key Laboratory of Medical Neurobiology, MOE Frontier Science Center for Brain Research and Brain-Machine Integration, School of Brain Science and Brain Medicine, Zhejiang University, 310058 Hangzhou, China; 3 Department of Anesthesiology, Technische Universität München/Klinikum Rechts der Isar, 81675 Munich, Germany; 4 Institute of Neuroscience and Department of Neurology of the Second Affiliated Hospital, Zhejiang University School of Medicine, 310058 Hangzhou, China

**Keywords:** Hippocampus, Molecular neuroscience

## Abstract

The cell adhesion molecule nectin3 and its presynaptic partner nectin1 have been linked to early-life stress-related cognitive disorders, but how the nectin1-nectin3 system contributes to stress-induced neuronal, circuit, and cognitive abnormalities remains to be studied. Here we show that in neonatally stressed male mice, temporal order and spatial working memories, which require the medial entorhinal cortex (MEC)-CA1 pathway, as well as the structural integrity of CA1 pyramidal neurons were markedly impaired in adulthood. These cognitive and structural abnormalities in stressed mice were associated with decreased nectin levels in entorhinal and hippocampal subregions, especially reduced nectin1 level in the MEC and nectin3 level in the CA1. Postnatal suppression of nectin1 but not nectin3 level in the MEC impaired spatial memory, whereas conditional inactivation of nectin1 from MEC excitatory neurons reproduced the adverse effects of early-life stress on MEC-dependent memories and neuronal plasticity in CA1. Our data suggest that early-life stress disrupts presynaptic nectin1-mediated interneuronal adhesion in the MEC-CA1 pathway, which may in turn contribute to stress-induced synaptic and cognitive deficits.

## Introduction

Cell adhesion molecules (CAMs) modulate the formation, maintenance, and remodeling of interneuronal connections, including synapses and extra-synaptic adherens junctions [1–3]. Nectins (nectin1–4) are a family of Ca^2+^-independent immunoglobulin-like CAMs [4]. Among the four members, presynaptic nectin1 and postsynaptic nectin3 mediate heterophilic adhesion at both synaptic and extra-synaptic sites during early development yet selectively at adherens junctions after the neonatal period, which plays important roles in the formation of synapses [5] and adherens junctions [6].

The structure and function of synapses undergo dynamic and experience-dependent reorganizations throughout life [1, 7]. Highly stressful experiences, especially those occurring in critical developmental periods, can lastingly disrupt synaptic plasticity and memory and increase the risk for neuropsychiatric disorders [8–10]. Recently, the involvement of nectin1 and nectin3 in cognition and stress-related cognitive disorders has been reported [11]. After contextual fear conditioning, nectin1 protein level in the synaptic fraction of the ventral hippocampus is upregulated, which in turn consolidates fear memory [12]. Emerging evidence also shows that repeated exposure to severe stress in the early postnatal period or later life stages alters nectin1 and nectin3 levels in the prefrontal [13], hippocampal [14–18], and parahippocampal [19] regions. Moreover, postnatal suppression of hippocampal nectin3 leads to spatial memory deficits in adulthood [20]. These findings highlight the modulation of stress effects by nectin3 in the hippocampus.

Hippocampal neurons receive both intrinsic and extrinsic inputs. Although the postsynaptic CAM nectin3 in the hippocampus has been linked to the effects of stress, it is unknown whether nectin1 expression in subpopulations of presynaptic neurons can be affected by early-life stress as well. More importantly, whether nectin1 in these neurons modulates stress-induced synaptic and cognitive impairments remains to be investigated. Considering that the medial entorhinal cortex (MEC) constitutes a major gateway between the neocortex and the hippocampus [21], it is possible that early-life stress targets nectin1 in MEC neurons to influence synaptic plasticity and memory.

In the current study, we evaluated the influence of early postnatal stress on memory performance, neuronal plasticity, and nectin expression level in medial entorhinal and hippocampal subregions during development and adulthood. Using both viral-mediated knockdown and conditional transgenic inactivation of nectin1, we further examined the potential role of MEC nectin1 in early-life stress-induced memory loss and neuronal remodeling. Our findings suggest that a reduction of MEC nectin1 level is associated with early-life stress-induced dendritic maldevelopment in CA1 pyramidal neurons and MEC-dependent memory deficits.

## Materials and methods

### Animals

C57BL/6N mice (SLAC Laboratories, Shanghai, China), CMV-Cre mice (stock number 006054, Jackson laboratory, Bar Harbor, ME, USA), and *nectin1*^*loxP/loxP*^ mice (Shanghai Model Organisms Center, Inc., Shanghai, China) were purchased and bred at the Laboratory Animal Center of Zhejiang University. *Nectin1*^*loxP/loxP*^ mice with exons 3 and 4 of the *nectin1* gene flanked by a pair of *loxP* sites were generated on the C57BL/6J background using standard CRISPR/Cas9 methods at Shanghai Model Organisms Center. For validation, female *nectin1*^*loxP/loxP*^ mice were crossed in-house with male CMV-Cre mice that expressed Cre in all tissues.

Adult mice were group-housed (3-4 per cage) under a 12:12 light:dark cycle (lights on from 8:00 to 20:00), controlled temperature (22 ± 2 °C) and humidity (50 ± 10%) conditions with free access to food and water, and were randomly assigned to each group. At 1 week before behavioral testing, mice were individually housed. The experiments were approved by the Animal Advisory Committee at Zhejiang University and performed in compliance with the National Institute of Health’s Guide for the Use and Care of Laboratory Animals.

### Experimental design

**Fig. 1:**
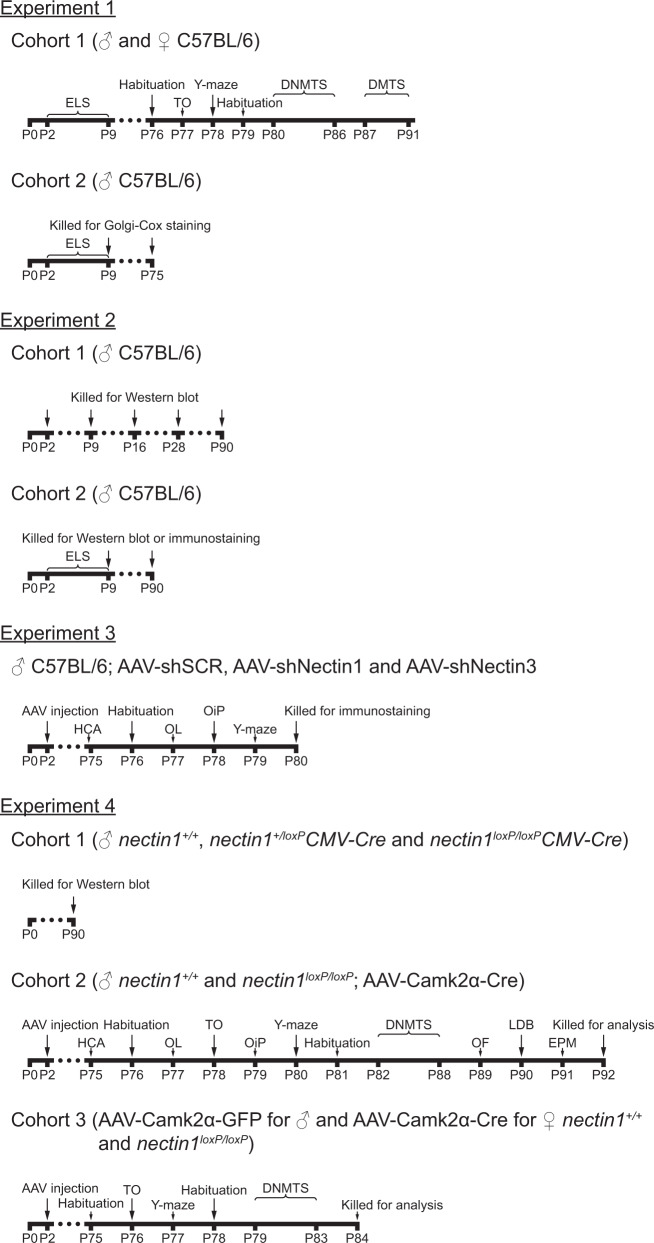
Schematic of experimental design. AAV adeno-associated virus; CMV cytomegalovirus; DMTS delayed match-to-sample; DNMTS delayed non-match-to-sample; ELS early-life stress; EPM elevated plus maze; HCA home cage activity; LDB light-dark box; OF open field; OiP object-in-place; OL object location; P postnatal day; TO temporal order.

The design of the experiments was summarized in Fig. 1. Experiment 1 examined the effects of early-life stress on temporal order and spatial working memories in adult male and female mice. Moreover, the short- and long-term impact of early-life stress on the morphology of CA1 pyramidal neurons in male mice was investigated. In experiment 2, hippocampal nectin1 and nectin3 protein levels across development and early adulthood, as well as the effects of early-life stress on nectin expression in entorhinal and hippocampal regions in male mice, were evaluated. Experiment 3 screened the influences of postnatal nectin1 or nectin3 knockdown in the MEC on memory performance in adult male mice. Experiment 4 further examined the effects of postnatal entorhinal nectin1 deletion on behavioral performance and structural plasticity in both adult male and female mice.

For group allocation, simple randomization was used. The sample size (*n*) was chosen based on published work and is comparable to that in our previous studies [22, 23]. During behavioral and image analyses, investigators were blind to the experimental conditions.

### Early-life stress procedure

The limited bedding and nesting paradigm was performed as detailed previously [15, 24]. On the morning of postnatal day 2 (P2), litters were culled to 6–8 pups with equal numbers of males and females whenever possible. Dams in the “control” cages were provided with sufficient nesting material (4.8 g of Nestlets, Indulab, Gams, Switzerland) and 500 ml of sawdust bedding, while those in the “stress” cages were provided with a limited quantity of nesting material (1.2 g of Nestlets) that was placed on an aluminum mesh platform (McNichols, Tampa, FL, USA). All litters remained undisturbed until the morning of P9 when the stress procedure ended. Mice were weaned on P28 and group-housed.

### Viral injection into the neonatal MEC

The following adeno-associated virus (AAV) vectors packaged by Obio Technology (Shanghai, China) were used in this study: AAV2/8-CMV-bGlobin-EGFP-H1-shScrambled (abbreviated as AAV-shSCR, 3.5 × 10^12^ viral genomes/ml), AAV2/8-CMV-bGlobin-EGFP-H1-shNectin1 (abbreviated as AAV-shNectin1, 5.98 × 10^12^ viral genomes/ml), and AAV2/8-CMV-bGlobin-EGFP-H1-shNectin3 (abbreviated as AAV-shNectin3, 3.9 × 10^12^ viral genomes/ml). These AAVs have been validated previously [20, 23]. pENN.AAV9.Camk2.HI.GFP-Cre.WPRE.SV40 (abbreviated as AAV-Camk2α-Cre) was a gift from James M. Wilson (Addgene viral prep #105551-AAV9; http://n2t.net/addgene:105551; RRID: Addgene_105551), and AAV2/8-Camk2α-EGFP-WPRE-hGH-polyA (abbreviated as AAV-Camk2α-GFP, 5.6 × 10^12^ viral genomes/ml; BrainVTA Biotechnology, Wuhan, China) was used as the control.

On the morning of P2, pups were consecutively collected and placed on a heating pad in another room. Pups were paw pad-tattooed according to a 4-paw identification scheme by the NEO-9 Neonate Rodent Tattooing System (Animal Identification and Marking Systems, Budd Lake, NJ, USA) to facilitate identification. Pups were then quickly anesthetized by an isoflurane-soaked cotton ball and secured in a stereotaxic frame (catalog number 68018, RWD Life Science, Shenzhen, China). The anesthesia was maintained using isoflurane-O_2_ (1–1.5:100) inhalation. AAV (0.1 μl per hemisphere) solutions were bilaterally delivered to the MEC via a glass micropipette connected to a Nanoject III microinjector (Drummond Scientific, Broomall, PA, USA) over a 5-min period. The injection site was determined according to an atlas of the developing mouse brain [25] and our pilot experiments. After microinjection, the micropipette was left on site for 5 min. Pups were then placed back on the heating pad until they resumed movement and then transferred to their home cages.

### Behavioral testing

Behavioral tests were performed during the light phase (9:00–19:00) and analyzed by ANY-maze 6.3 software (Stoelting, Wood Dale, IL, USA) as detailed previously [22, 26]. The testing apparatus was thoroughly cleaned with 70% ethanol followed by distilled water between tests.

### Object recognition tasks

Object recognition tasks were performed in the open field arena (50 × 50 × 50 cm^3^, 10 lux). At 24 h before the acquisition phase, mice were habituated to the testing environment without objects or spatial cues. During testing, objects were placed adjacent to the corners of the arena, 5 cm from the walls. The habituation, acquisition, and retrieval trials lasted 10 min each. The time exploring each object and the discrimination index (DI) in the retrieval phase were quantified.

The temporal order task consisted of three trials separated by 60 min of intertrial intervals (ITIs) without spatial cues. In acquisition phases 1 and 2, two cubes and two cylinders were presented respectively. In the retrieval phase, a “remote” object (cube) and a “recent” one (cylinder) were presented. DI was calculated as 100% × (time with the remote object-time with the recent object)/time with both objects.

The object location task consisted of two trials separated by an ITI of 24 h with prominent spatial cues. In the acquisition phase, two identical circular cones were presented. In the retrieval phase, a non-displaced object and a relocated one were presented. DI was calculated as 100% × (time with the relocated object-time with the stationary object)/time with both objects.

The object-in-place task consisted of two trials separated by an ITI of 30 min with prominent spatial cues. In the acquisition phase, four objects with distinct shapes were presented. In the retrieval phase, two of the objects exchanged positions. DI was calculated as 100% × (time with the relocated objects-time with the stationary objects)/time with all objects.

### Y-maze spontaneous alternation task

The Y-maze apparatus was made of gray polyvinyl chloride with three symmetrical arms (30 × 10 × 15 cm^3^, 10 lux). Prominent extra-maze spatial cues were provided. Mice were placed at the end of one arm and allowed to explore for 5 min. Three consecutive choices of all three arms were recorded as an alternation. The percentage of spontaneous alternation was determined by dividing the total number of alternations by the total number of choices minus 2.

### Delayed non-match/match-to-sample tasks

The body weight of mice was adjusted to 85–90% of their starting weight before testing. Mice were habituated in the Y-maze for three trials. In the first habituation trial, mice learned to collect dustless precision pellets (45 mg, F0299, Bio-Serv, Frenchtown, NJ, USA) from the maze center and the two food cups at the end of two choice arms. In the following two trials, food pellets were only present in the food cups. After habituation, the delayed non-match-to-sample (DNMTS) and delayed match-to-sample (DMTS) tasks were carried out consecutively in the Y-maze as previously described with minor modifications [26].

In the DNMTS task, mice were tested in 4 trials per day for 7 days. The ITIs were 20 min, and each trial consisted of a sample run and a choice run. In the sample run, both choice arms were baited but one of them was blocked in a pseudo-random manner. After collecting the food reward, mice were placed back to the starting arm for 5 s, during which the block was removed from the choice arm. In the choice run, a correct choice was scored when mice explored the unvisited arm in the sample run and consumed the food reward. Mice that entered the previously visited choice arm were confined to the arm, allowed to explore the empty food cup for 10 s, and transferred to their home cages. The percentage of correct choices in all trials each day was calculated.

The DMTS task lasted for 5 days with 4 trials per day that were separated by 20 min of ITIs. In the choice run, mice were reinforced for visiting the same arm as in the sample run. A correct choice was scored when mice explored the previously visited arm and consumed the food reward. The percentage of correct choices was calculated.

### Home cage activity assessment

Home cage locomotion and exploration were monitored for 24 h using a custom-made counter as previously described [27]. Activity counts in the light and dark phases were recorded separately.

### Open field test

Mice were tested in the open field arena (50 × 50 × 50 cm^3^, 30 lux) made of gray polyvinyl chloride. The time spent in the center zone (25 × 25 cm^2^) and total distance traveled were recorded for 10 min.

### Light-dark box test

Mice were placed in the dark chamber (15 × 20 × 25 cm^3^, 10 lux) that was connected to the brightly illuminated chamber (30 × 20 × 25 cm^3^, 650 lux) by a tunnel. The time spent in the light chamber was recorded during the 5-min test.

### Elevated plus maze test

The elevated plus maze was made of gray polyvinyl chloride with two opposing open arms (30 × 5 × 0.5 cm^3^, 40 lux) and two opposing enclosed arms (30 × 5 × 15 cm^3^, 10 lux) connected by a central platform (5 × 5 cm^2^). Mice were placed on the central platform and allowed to explore for 5 min. The percentage of time in open arms was calculated as 100% × time in open arms/time in all arms.

### Golgi–Cox staining and the analysis of dendrites and spines

Golgi–Cox staining was performed with the FD Rapid GolgiStain^TM^ Kit (FD NeuroTechnologies, Ellicott, MD, USA) as recently described [22]. In brief, neonatal and adult brains were immersed in the Golgi–Cox solution for 9 and 14 days, respectively, and then transferred to 30% sucrose solution for 2–5 days in the dark. Serial coronal sections (120 µm) were cut using a Leica VT1000S vibratome (Leica, Wetzlar, Germany), mounted on Superfrost plus slides (Thermo Scientific, Rochester, NY, USA), stained, and coverslipped.

Z-stack bright-field images (4140 × 3096 pixel^2^ for dendrites and 2070 × 1548 pixel^2^ for spines) were obtained using a DP72 camera fitted to an Olympus BX61 microscope (Olympus, Tokyo, Japan) equipped with an automated ProScan stage (Prior Scientific, Rockland, MA, USA). For dendrite analysis, fully impregnated CA1 pyramidal neurons (6–8 neurons per mouse) were scanned using a 40× objective with a z-step size of 1 μm. Sholl analysis was performed to measure total dendritic length and the number of intersections at concentric circles (10 μm apart) by the NeuronStudio software [28]. Apical and basal dendritic features were extracted separately. The border between the stratum radiatum and the stratum lacunosum-moleculare (SLM) of CA1 was identified by adjusting the brightness and contrast of the images. The percentage of dendritic invasion to CA1 SLM was calculated as 100% × total length of dendritic segments in SLM/total length of apical dendrites. For spine analysis, intact dendritic branches (8 branches from different neurons per mouse) in CA1 SLM were scanned using a 60× oil-immersion objective with a z-step size of 0.5 μm. Dendritic protrusions were categorized as thin spines, mushroom spines, stubby spines, and filopodia using NeuronStudio based on established criteria [29]. Spine density was expressed as the number of spines per 10 μm of dendrite. For all morphological analyses, results were first averaged by neurons from an animal and then averaged by animals in a group.

### Immunostaining and image analysis

Mice were anesthetized with sodium pentobarbital (neonatal: 100 mg/kg, adult: 200 mg/kg; i.p.) and transcardially perfused with 0.9% saline followed by 4% buffered paraformaldehyde. Following post-fixation and cryoprotection, serial horizontal sections (30 or 40 μm thick) were prepared through the hippocampus and MEC (Bregma −2.16 to −4.12 mm) using a Leica CM1950 cryostat (Leica). Primary rabbit anti-nectin1 (1:1000, sc-28639, Santa Cruz Biotechnology, Santa Cruz, CA, USA), rabbit anti-nectin3 (1:1000, ab63931, Abcam, Cambridge, UK), and guinea pig anti-synaptophysin (1:500, 101004, Synaptic Systems, Göttingen, Germany) antibodies were used.

For immunofluorescence, after incubation with primary antibodies at 4 °C (overnight) and rinsing, free-floating sections were labeled with a donkey anti-rabbit Alexa Fluor 488-conjugated secondary antibody (1:2000, A-21206, Invitrogen, Carlsbad, CA, USA) at room temperature (3 h). For double-labeling immunofluorescence, a donkey anti-guinea pig Alexa Fluor 488-conjugated antibody (1:2000, 706-545-148, Jackson ImmunoResearch, West Grove, PA, USA) and a donkey anti-rabbit Alexa Fluor 594-conjugated antibody (1:2000, A-21207, Invitrogen) were used. Sections were rinsed, mounted onto slides (Citotest, Haimen, China), and coverslipped with Vectashield (Vector Laboratories, Burlingame, CA, USA). For immunohistochemistry, sections were treated with 3% hydrogen peroxide (10 min) followed by 1% normal goat serum (1 h), and were labeled with primary antibodies at 4 °C (overnight). The next day, sections were rinsed and incubated with a biotinylated goat anti-rabbit secondary antibody (Zhongshan Golden Bridge Biotechnology, Beijing, China) at room temperature (2 h). After rinsing, the 3,3’-Diaminobenzidine Horseradish Peroxidase Color Development Kit (Zhongshan Golden Bridge) was used for staining. The sections were then transferred onto slides, dried and coverslipped.

Immunofluorescent images (1600 × 1600 or 1024 × 1024 pixel^2^) were obtained with an Olympus IX81-FV1000 laser scanning confocal microscope equipped with a 10× (NA 0.40) and a 60× oil-immersion (NA 1.42) objectives using the Kalman filter under identical settings for laser power, photomultiplier gain and offset. To visualize synaptophysin- or nectin1-immunoreactive puncta, z-stack images with a step size of 0.5 μm were deconvolved by Huygens Essential 21.10 software (Scientific Volume Imaging, Hilversum, The Netherlands) using automatic settings. Immunohistochemical images (4140 × 3096 pixel^2^) were acquired at ×100 with the Olympus BX61 microscope (Olympus). All image data were analyzed with the ImageJ 1.52e software (National Institute of Health, Bethesda, MD, USA). Relative protein levels were determined by the differences in optical density values between the region of interest and corpus callosum from the same section that was set as the background. Results were normalized by taking the mean value of the control group as 100%.

### Western blot

Western blot was performed as described previously [15]. MEC and hippocampal tissue from both hemispheres was dissected and homogenized in ice-cold lysis buffer and centrifuged at 10,000 rpm at 4 °C (20 min). Protein concentrations were determined using a bicinchoninic acid protein assay kit (Pierce, Rockford, IL, USA). Samples containing 20 μg of protein were resolved by 10% sodium dodecyl sulfate-polyacrylamide gels and transferred onto polyvinylidene difluoride membranes (Millipore, Bedford, MA, USA). Membranes were then labeled with primary rabbit anti-nectin1 (1:2000, Santa Cruz), rabbit anti-nectin3 (1:2000, Abcam), mouse anti-actin (1:10,000, E021020-01, EarthOx, Millbrae, CA, USA), mouse anti-beta-tubulin (1:10,000, E021040-01, EarthOx), or mouse anti-glyceraldehyde-3-phosphate dehydrogenase (GAPDH; 1:5000, E021010-01, EarthOx) antibodies at 4 °C (overnight). Following incubation with horseradish peroxidase-conjugated secondary antibodies (1:2000, Sigma-Aldrich, St Louis, MO, USA) at room temperature (3 h), bands were visualized using an enhanced chemiluminescence system (Pierce) and quantified by densitometry (Quantity One 4.2, Bio-Rad, Hercules, CA, USA). Each assay was repeated three times. Results were normalized by taking the mean value of the control group as 100%.

### Statistical analysis

SPSS 22.0 (SPSS, Chicago, IL, USA) and GraphPad Prism 9.0 (GraphPad Software Inc., San Diego, CA, USA) were used to perform statistical analyses. For between-group comparisons, two-tailed unpaired Student’s *t* test were applied. Pooled-variance *t* test was used when equal variances were assumed, while a separate-variance *t* test was applied when the variance was not equal. For multiple group comparisons, data were analyzed by analysis of variance (ANOVA) followed by Tukey’s or Dunnett’s post hoc test when appropriate. The DNMTS and DMTS data, nectin immunoreactivity along the dorsoventral axis of the hippocampus, and the number of dendritic intersections at concentric circles were analyzed by one-way repeated-measures ANOVA. Statistical outliers with values that fell beyond two s.d.’s from the mean were excluded from the analysis [23, 30]. Data are reported as mean ± SEM. Statistical significance was defined at *p* < 0.05. Detailed statistics are provided in Supplementary Table S1.

## Results

### Early-life stress impaired MEC-dependent memories in a gender-specific manner

**Fig. 2:**
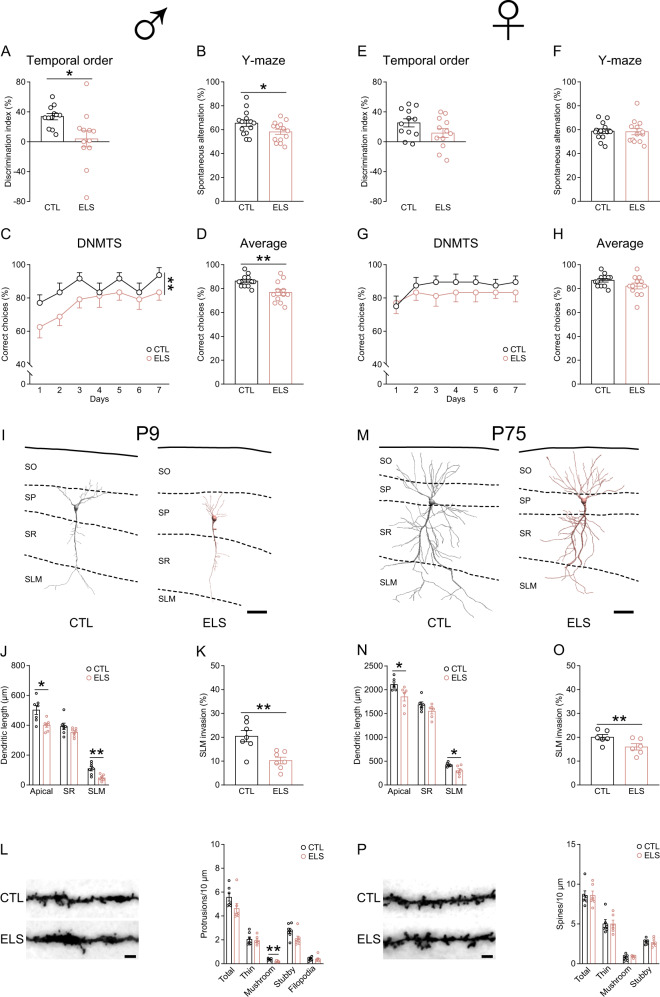
Effects of early-life stress on temporal order memory, spatial working memory, and morphology of CA1 pyramidal neurons. **A** Adult male mice with early-life stress (ELS) exposure showed impaired performance in the temporal order task compared to controls (CTL). **B** Postnatally stressed male mice showed impaired performance in the Y-maze spontaneous alternation task. In the Y-maze delayed non-match-to-sample (DNMTS) task, postnatally stressed male mice showed impaired acquisition and made more errors over the training sessions (**C**) and on average (**D**) than control mice. *n* = 12 mice (**A**, **C**, and **D**) or 14 mice (**B**) per group. Early-life stress did not affect the performance of adult female mice in the temporal order task (**E**), the Y-maze spontaneous alternation task (**F**), nor the DNMTS task (**G**, **H**). *n* = 12 mice per group for (**E**, **G**, and **H**). 14 CTL and 13 ELS mice were included in **F**. **I** Representative reconstructions of CA1 pyramidal neurons from 9-day-old control and stressed male pups. P, postnatal day; SO, stratum oriens; SP, stratum pyramidale; SR, stratum radiatum; SLM, stratum lacunosum-moleculare. **J** After exposure to early postnatal stress, total length of apical dendrites and the length of dendritic segments in CA1 SLM were significantly shorter than those in controls. **K** The percentage of dendritic extension to SLM was reduced in stressed pups. **L** Left: representative images of dendritic segments in the SLM of CA1. Right: analysis of dendritic protrusion density, showing that the density of mushroom spines was reduced in stressed pups. *n* = 7 mice per group for **J**–**L**. **M** Representative reconstructions of CA1 pyramidal neurons from adult control and ELS mice. **N** Compared to control mice, total length of apical dendrites and the length of dendritic segments in CA1 SLM were reduced in adult ELS mice. **O** The invasion of apical dendrites to CA1 SLM was reduced in adult ELS mice. **P** Left: representative images of dendritic segments in the SLM of CA1. Right: no difference in dendritic spine density was found between groups. *n* = 6 mice per group for **N**–**P**. Scale bars are 50 µm for neuronal reconstructions and 2 µm for dendrite images. *p < 0.05; **p < 0.01. For this and subsequent figures, detailed statistics are provided in Supplementary Table S1.

We first examined the behavioral effects of early postnatal stress, especially the stress effects on temporal order memory and spatial working memory, in adult male and female mice. In the temporal order task, control and stressed male mice explored the objects similarly in the acquisition phases (Fig. S1A and B). In the retrieval phase, stressed mice showed markedly impaired memory performance compared to control mice (Figs. 2A and S1C). In the Y-maze spontaneous alternation task, stressed mice had lower spontaneous alternation rates (Fig. 2B) but more arm entries (Fig. S1D) than the controls. To further evaluate spatial working memory, we employed the DNMTS and DMTS tasks. Stressed mice made more incorrect choices than controls in the DNMTS task (Fig. 2C and D), indicative of impaired spatial working memory, whereas both groups showed comparable performance in the DMTS task (Fig. S1E and S1F). By contrast, adult female mice with adverse early-life experiences exhibited normal performance in the temporal order task (Figs. 2E and S1G–S1I) and Y-maze tasks (Figs. 2F–H and S1J–L). These results indicate that the negative influence of early-life stress on MEC-dependent memories is gender-specific.

### Early-life stress induced subregion-specific dendritic remodeling of CA1 pyramidal neurons

To uncover the cellular basis of early postnatal stress-induced memory deficits, we evaluated the immediate and lasting effects of stress on the morphological development and plasticity of hippocampal CA1 pyramidal neurons. On P9 when the stress procedure was ended, the total length and complexity of apical but not basal dendrites of CA1 neurons were significantly reduced in stressed pups (Figs. 2I, J, and S1M-S1O). Notably, the stress effects on dendritic development were more prominent in the SLM of CA1, which receives extra-hippocampal inputs from entorhinal regions including MEC (Fig. 2J, K). In stressed pups, the density of mushroom spines in CA1 SLM was also reduced, while the density of total protrusions and other protrusion subtypes remained unchanged (Fig. 2L). Early-life stress-induced dendritic abnormalities of CA1 neurons persisted to young adulthood, as shown by reduced length of apical dendrites in SLM in neonatally stressed mice (Fig. 2M–O and S1P–R). In comparison, the density of all spines and spine subtypes were comparable between groups (Fig. 2P). These data revealed that early-life stress hampered the development and remodeling of CA1 pyramidal neurons in a layer-specific and possibly input-dependent manner.

### Early-life stress lastingly suppressed nectin levels in entorhinal and hippocampal subregions

**Fig. 3:**
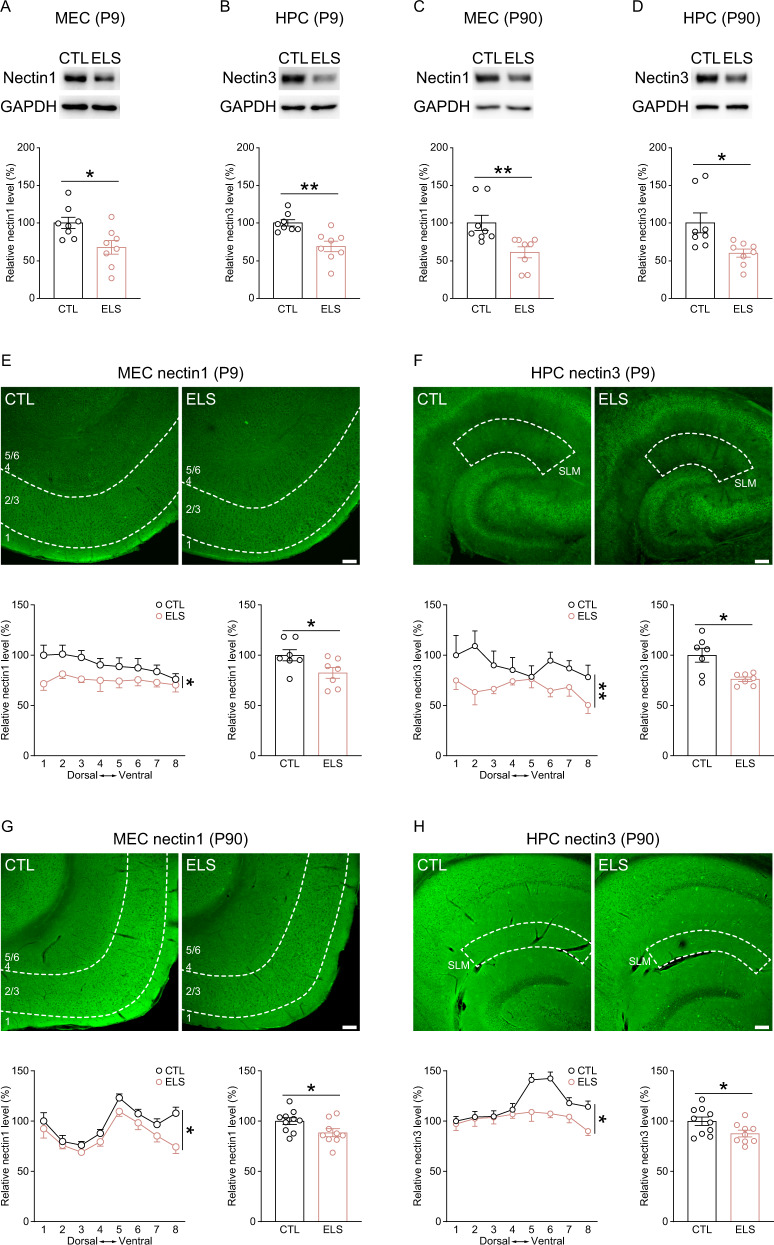
Short-term and long-lasting effects of early-life stress on nectin protein levels in medial entorhinal and hippocampal subregions. Compared to controls, stressed pups showed reduced protein levels of nectin1 in the medial entorhinal cortex (MEC; **A**) and nectin3 in the hippocampus (HPC; **B**). In adulthood, a history of postnatal stress exposure led to a reduction of MEC nectin1 (**C**) and hippocampal nectin3 (**D**) protein levels. For **A**–**D**, representative immunoblots were shown, and eight mice per group were included. Top panels: representative images showing the immunostaining of the presynaptic CAM nectin1 in the MEC (**E**) and the postsynaptic CAM nectin3 in the hippocampus (**F**) in control and stressed pups. Bottom: analysis of nectin1 immunoreactivity in MEC layers 2/3 and nectin3 immunoreactivity in the SLM of CA1 along the dorsoventral axis, showing that nectin1 level in the MEC and nectin3 level in the SLM of CA1 were reduced in stressed pups compared to controls. *n* = 7 mice per group. Top panels: representative images showing nectin1 immunostaining in the MEC (**G**) and nectin3 immunostaining in the hippocampus (**H**) in adult mice with or without early-life stress experience. Bottom: early-life stress lastingly suppressed nectin1 level in MEC layers 2/3 and nectin3 level in CA1 SLM. Ten CTL and nine ELS mice were included. Scale bars are 100 µm. *p < 0.05; **p < 0.01.

Considering the role of the nectin system in experience-dependent synaptic plasticity and stress-related disorders [4, 11], we examined hippocampal nectin protein levels during development and early adulthood, and evaluated the impact of neonatal stress on nectin levels in medial entorhinal and hippocampal subregions. Hippocampal nectin1 and nectin3 protein levels showed developmental fluctuations, with relatively high levels during postnatal weeks 2–4 than on P2 and adulthood (Fig. S2A and B). Immediately after stress ended, entorhinal nectin1 and hippocampal nectin3 protein levels were significantly reduced (Fig. 3A, B). In adulthood, MEC nectin1 and hippocampal nectin3 levels in postnatally stressed mice remained lower than control mice (Fig. 3C, D). Consistent with immunoblot results, immunostaining revealed that both nectin1 and nectin3 immunoreactivity was reduced in entorhinal and hippocampal subregions in stressed pups (Fig. S2C and D), with a more pronounced reduction of nectin1 level in MEC layers 2/3 (Fig. 3E) and nectin3 level in CA1 SLM (Fig. 3F). Similarly, nectin1 and nectin3 immunoreactivity in specific layers of MEC and hippocampus, especially nectin1 level in MEC layers 2/3 and nectin3 level in CA1 SLM, was lower in stressed adult mice than in control mice (Figs. 3G, H, S2E, and S2F). Although the reduction of nectin1 and nectin3 levels relatively lacked subregion specificity, these results raise the possibility that the nectin1-nectin3 system in the MEC-CA1 pathway is susceptible to early-life stress, and suggest that dysregulated MEC nectin1 level may participate in stress-induced cognitive and structural deficits.

### Postnatal knockdown of nectin1 but not nectin3 in the MEC impaired spatial memory in adulthood

**Fig. 4:**
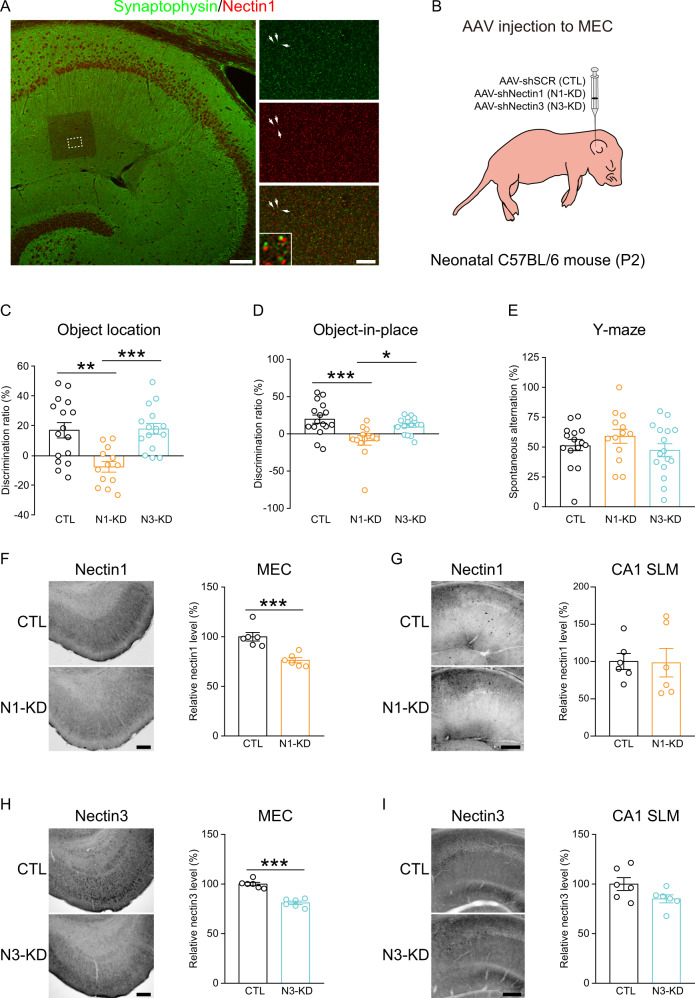
Subcellular localization of nectin1 in CA1 SLM and the effects of postnatal nectin knockdown in the MEC on spatial memory. **A** Left panel: a representative confocal image showing nectin1 and synaptophysin immunostaining in the adult hippocampus. The boxed region is zoomed in and separated by channels to the right. Scale bar is 100 µm. Right panels: Arrows indicate representative nectin1- or/and synaptophysin-immunoreactive puncta. As shown by the boxed image at the bottom, nectin1 was closely adjacent to synaptophysin. Scale bar is 10 µm. **B** Schematic showing viral microinjection into the MEC of neonatal pups on P2. Behavioral tests were performed in early adulthood. **C** Adult mice with postnatal knockdown of nectin1 (N1-KD) but not nectin3 (N3-KD) in the MEC showed impaired performance in the object location task, indicative of spatial memory deficits. **D** Postnatal knockdown of nectin1 but not nectin3 in the MEC also impaired performance in the object-in-place task. For **C**, **D** 16 CTL, 13 N1-KD and 16 N3-KD mice were included. **E** Neither nectin1 nor nectin3 knockdown in the MEC affected performance in the Y-maze spontaneous alternation task. 15 CTL, 13 N1-KD and 16 N3-KD mice were used. Validation of nectin1 knockdown in the MEC. In N1-KD mice, nectin1 immunoreactivity was specifically reduced in the MEC (**F**) but not the SLM of CA1 (**G**). Validation of nectin3 knockdown in the MEC. In N3-KD mice, nectin3 immunoreactivity was selectively reduced in the MEC (**H**) but not CA1 SLM (**I**). For **F**–**I**, six mice per group were included, and scale bars are 200 µm. *p < 0.05; **p < 0.01; ***p < 0.001.

Hippocampal nectin1 and nectin3 have been shown to preferentially localize on the pre- and post-synaptic sides respectively [5]. We found that in the SLM of CA1, although nectin1-immunoreactive puncta rarely overlapped with the pre-synaptic marker synaptophysin, these two molecules were closely adjacent to each other (Fig. 4A), indicating that nectin1 is present in presynaptic-like structures in CA1 SLM.

Considering the involvement of hippocampal nectin3 in stress-induced memory loss [14, 16, 20, 23, 31], we tested whether a downregulation of its presynaptic partner nectin1 in MEC neurons could mimic neonatal stress-induced spatial memory impairments. Since reduced nectin3 levels were also noticed in the adult MEC, the cognitive effects of MEC nectin3 knockdown were evaluated. We injected nectin1- or nectin3-knockdown virus to the neonatal MEC on P2 and screened the effects of nectin1 or nectin3 suppression on spatial memory performance in adulthood (Fig. 4B). In the object location task, adult mice with postnatal MEC nectin1 knockdown showed impaired object discrimination, indicative of spatial memory impairments (Figs. 4C and S3A). By contrast, mice with suppressed nectin3 level in the MEC had intact spatial memory. In the object-in-place task, nectin1 but not nectin3 knockdown mice also showed impaired performance (Figs. 4D and S3B). In the Y-maze spontaneous alternation task, no difference in spontaneous alternation rates nor total arm entries was observed among groups (Figs. 4E and S3C). In addition, neither nectin1 nor nectin3 knockdown affected home cage activity (Fig. S3D). We also validated the efficiency of virus-mediated knockdown. The downregulation of nectin1 or nectin3 protein level was restricted to the MEC, while nectin immunoreactivity in CA1 SLM remained unchanged (Fig. 4F–I). These data are complementary to previous reports on the role of hippocampal nectin3 in stress-induced memory loss, and suggest that nectin1 in the MEC also contributes to the adverse cognitive effects of early-life stress.

### Conditional deletion of nectin1 from the MEC mimicked early-life stress-induced memory loss and dendritic remodeling

**Fig. 5:**
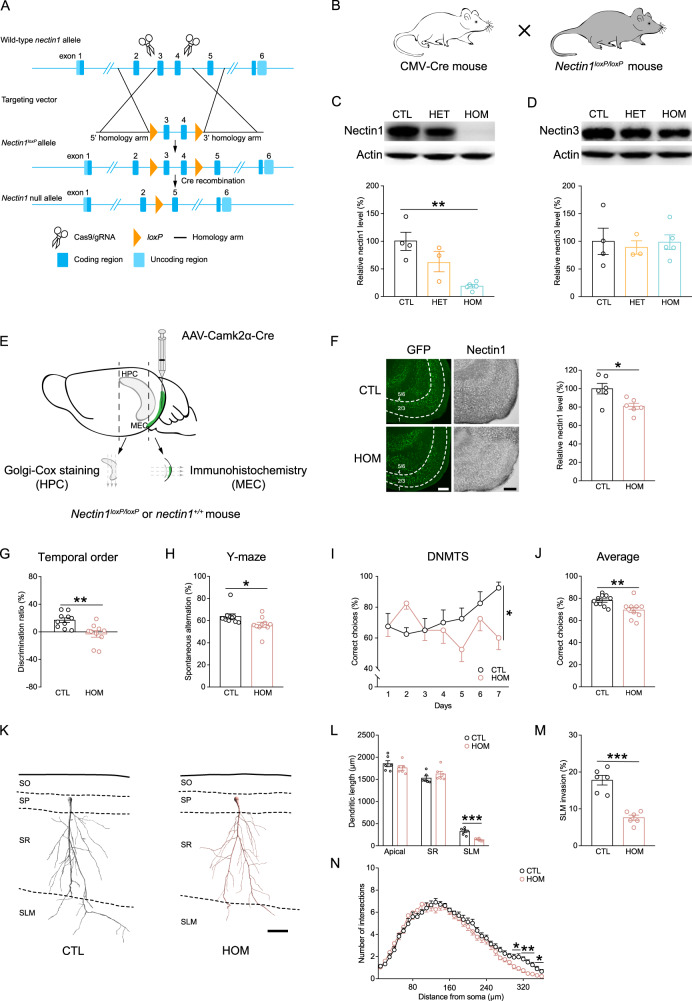
Effects of postnatal inactivation of nectin1 in MEC excitatory neurons on memory and structural plasticity of CA1 pyramidal neurons. **A** Strategy for Cre recombinase-mediated conditional inactivation of the *nectin1* gene using the CRISPR/Cas9 system. Partial restriction maps of the wild-type *nectin1* locus, targeting vector, *nectin1*^*loxP*^ allele, and mutant *nectin1* locus are shown. In the targeting vector, exons 3 and 4 of the *nectin1* gene were flanked by a pair of *loxP* sites. **B** To assess the efficiency and specificity of *nectin1* gene knockout, CMV-Cre mice that expressed Cre under the control of the cytomegalovirus promoter were crossed with *nectin1*^*loxP/loxP*^ mice. The protein levels of nectin1 and nectin3 in hippocampal tissues from wild-type control (CTL, *nectin1*^*+/+*^), heterozygous (HET, *nectin1*^*+/loxP*^*CMV-Cre*), and homozygous (HOM, *nectin1*^*loxP/loxP*^*CMV-Cre*) mice were examined. Hippocampal nectin1 level in HOM mice was markedly reduced compared to control mice (**C**), while hippocampal nectin3 level was similar among groups (**D**). 4 CTL, 3 HET, and 5 HOM mice were used. **E** Illustration of the strategy to microinject AAV-Camk2α-Cre to the MEC of *nectin1*^*+/+*^ (CTL) or *nectin1*^*loxP/loxP*^ (HOM) pups on P2. After behavioral testing in adulthood, coronal sections through the hippocampus were prepared for Golgi-Cox staining, while horizontal sections through the MEC were prepared for immunohistochemistry. **F** Left: representative images showing the spread of AAV as revealed by green fluorescent protein (GFP) and nectin1 immunostaining in the MEC of control and HOM mice. Right: MEC nectin1 immunoreactivity was reduced in HOM mice. *n* = 6 mice per group. Compared to wild-type controls, HOM mice showed impaired object discrimination in the temporal order task (**G**), and had lower alternation rates (**H**) and more incorrect choices over the training sessions (**I**) and on average (**J**) in the Y-maze spontaneous alternation and DNMTS tasks. *n* = 10 mice per group. **K** Representative reconstructions of CA1 pyramidal neurons from control and HOM mice. Note that only apical dendrites were reconstructed and analyzed. Compared to control mice, the length of dendritic segments in CA1 SLM (**L**) and the percentage of dendritic invasion to SLM (**M**) were reduced in HOM mice, and the negative effects were more pronounced at 300–360 µm from the soma (**N**). *n* = 6 mice per group. Scale bars are 200 µm for fluorescent and immunostaining images and 50 µm for neuronal reconstructions. *p < 0.05; **p < 0.01; ***p < 0.001.

To further dissect the role of MEC nectin1 in early-life stress-induced memory loss and structural abnormalities, we generated a novel *nectin1*^*loxP/loxP*^ mouse line using the CRISPR/Cas9 system (Fig. 5A). By crossing *nectin1*^*loxP/loxP*^ mice with a mouse line that expressed Cre recombinase under the control of the cytomegalovirus (CMV) promoter in all tissues, the efficiency and specificity of *nectin1* gene knockout were examined (Fig. 5B). In homozygous (HOM) *nectin1*^*loxP/loxP*^*CMV-Cre* mutants, nectin1 protein level in the hippocampus was markedly decreased compared to control mice (Fig. 5C), whereas hippocampal nectin3 levels remained unchanged (Fig. 5D).

For postnatal inactivation of nectin1 in MEC excitatory neurons, an AAV expressing Cre recombinase and green fluorescent protein (GFP) under the control of the Camk2a promoter (AAV-Camk2α-Cre) was microinjected to the MEC of HOM mice and wide-type controls on P2. Behavioral tasks were performed in young adulthood, after which the brains were processed for structural analysis (Fig. 5E). As validated by immunostaining, nectin1 level in the adult MEC was significantly downregulated in HOM mice (Fig. 5F). In the acquisition phases of the temporal order task, control and HOM mice explored the objects comparably (Fig. S4A). However, HOM mice failed to discriminate the remote and the recent objects and performed worse than controls in the retrieval phase of the temporal order task (Figs. S4A and 5G). In the retrieval phase of the object location and the object-in-place tasks, HOM mice also showed impaired memory performance compared to control mice (Fig. S4B–E). In the Y-maze spontaneous alternation task, HOM mice had lower spontaneous alternation rates (Fig. 5H) but traveled longer distances (Fig. S4F) than control mice. In the Y-maze DNMTS task, HOM mice made more incorrect choices than control mice (Fig. 5I, J). As evidenced by the similar performance in the open field, light-dark box, and elevated plus maze tests between groups (Fig. S4G–J), nectin1 deletion in the MEC did not affect anxiety-related behaviors.

We also injected AAV-Camk2α-GFP that did not express Cre into the MEC of male wild-type and HOM mice on P2. In adulthood, wild-type and HOM mice injected with this control AAV did not differ in temporal order memory (Fig. S5A and B) and spatial working memory (Fig. S5C–E). As validated by immunostaining, both groups showed similar levels of nectin1 in the MEC (Fig. S5F), confirming that the control AAV did not induce nectin1 deletion. Moreover, we examined the effects of postnatal entorhinal nectin1 deletion on cognitive performance in adult female mice. Female HOM mice exhibited comparable temporal order and spatial working memories to wild-type mice (Fig. S5G–K), although MEC nectin1 protein level in these mice was significantly reduced (Fig. S5L). Taken together, postnatal deletion of MEC nectin1 in male but not female mice impaired MEC-dependent memories, which mimicked the cognitive effects of early postnatal stress.

We further performed Golgi staining and analyzed the morphology of apical dendrites of CA1 pyramidal neurons in male control and HOM mice (Fig. 5K). Although the total length of apical dendrites was comparable between groups, the length and complexity of dendritic segments in CA1 SLM were significantly reduced in HOM mice compared to control mice (Fig. 5L–N). Thus, postnatal deletion of MEC nectin1 could also reproduce the stress effects on the structural plasticity of CA1 pyramidal neurons.

## Discussion

In this study, we revealed nectin1 as a critical presynaptic CAM that potentially contributed to the adverse effects of early-life stress on the development and function of the medial entorhinal-hippocampal circuit. In adult male mice with neonatal stressful experiences, impairments of MEC-dependent memories and input-specific structural remodeling of CA1 pyramidal neurons were accompanied by persistent reduction of nectin1 and nectin3 levels in entorhinal and hippocampal subregions. Suppression of nectin1 but not nectin3 level in the neonatal MEC partially mimicked the cognitive impact of early-life stress, which is further corroborated by the evidence that conditional deletion of nectin1 from MEC excitatory neurons impaired cognitive performance and dendritic plasticity of CA1 neurons. These results also extend our understanding on the function of stress-related CAMs from cellular to circuit level.

Early-life experiences sculpt brain development and influence lifelong well-being [32]. Adverse early experiences may evoke cognitive deficits and markedly increase the risk for neuropsychiatric disorders [9, 10]. In rodent models of early-life stress, animals with neonatal stressful experiences consistently show impaired hippocampus-dependent learning and memory [33, 34]. Using the limited bedding and nesting paradigm that induces fragmented maternal care around the first postnatal week [24], we further showed that early-life stress impaired temporal order and spatial working memories that involve the MEC-CA1 pathway [35, 36]. Notably, impairments in MEC-dependent memories were only observed in stressed male but not female mice. In addition, females with postnatal entorhinal nectin1 deletion showed intact memory. Similar gender difference in memory performance following early-life stress has been reported previously [37]. Although glucocorticoid receptors have been implicated in the gender-specific effects of early-life stress [38], the mechanisms underlying such differences remain to be studied.

Early-life stress impairs neuronal development and remodeling, as exemplified by dendritic shrinkage and spine loss in hippocampal principal cells [39, 40]. In contrast to findings in rats where both apical and basal dendrites of CA1 neurons are affected [41, 42], we found a subregion-specific effect of early-life stress on dendritic morphology in the mouse CA1. The extension and branching of dendrites in the SLM of CA1, which receives extra-hippocampal inputs mainly from the medial and lateral entorhinal cortices, were hampered both immediately after stress ended and in adulthood. Consistent with our previous results [43], the density of dendritic protrusions in CA1 SLM remained mostly unchanged in postnatally stressed mice during development and adulthood, except for a transient decrease of mushroom spine density in the neonatal period. Taken together, these structural changes led to a significant reduction of total spine number per CA1 neuron, which may be responsible for cognitive deficits in stressed mice.

CAMs have long been hypothesized to modulate the effects of chronic stress on synaptic plasticity and memory [44]. Previous studies demonstrate the critical role of neural cell adhesion molecule [45], neuroligin-2 [46, 47], and nectin3 [16] in stress-induced cognitive deficits. Among these functionally characterized CAMs, nectin3 has been particularly linked to early postnatal stress [15, 17]. Early-life stress lastingly suppresses hippocampal nectin3 level, whereas stress-impaired hippocampus-dependent memory can be mimicked by nectin3 knockdown and rescued by nectin3 overexpression in hippocampal neurons [16, 23]. In this study, we found that the protein levels of nectin1 and nectin3 increased and peaked during the neonatal period and decreased afterward. Most importantly, early-life stress persistently reduced nectin1 and nectin3 levels in subregions of the hippocampus and specific layers of the MEC. In neonatally stressed mice, a prominent molecular change was the reduction of presynaptic nectin1 in MEC layer 2/3 and postsynaptic nectin3 in CA1 SLM, which was closely associated with SLM-specific dendritic abnormalities and deficits in MEC-dependent memories. As shown by a previous ultrastructural study in conventional nectin knockout mice, transgenic deletion of nectin1 or nectin3 reduces the density of adherens junctions but not synapses in the dentate gyrus-CA3 pathway [6]. These findings point to the possibility that disrupted nectin-mediated trans-neuronal adhesion may contribute to the adverse stress effects on the structure and function of the MEC-CA1 pathway.

We have recently shown that neonatal intraventricular injection of nectin3- but not nectin1-knockdown virus, which mainly infected the hippocampus, could impair object recognition memories and the structural plasticity of hippocampal neurons [20]. To dissect the involvement of medial entorhinal nectins in early-life stress-induced abnormalities, we first screened the cognitive effects of nectin1 or nectin3 knockdown in the MEC, and found that postnatal suppression of nectin1 but not nectin3 in the MEC impaired spatial memory in adulthood. By developing a novel conditional nectin1 knockout mouse line, we validated these findings and further showed that conditional deletion of nectin1 from MEC excitatory neurons mimicked the effects of early-life stress on MEC-dependent memories as well as dendritic plasticity of CA1 neurons. Although seemingly contradictory, our current and previous data reconcile with each other from a neural circuit perspective and take the synaptic localization of nectins into consideration. As a presynaptic CAM that is much more abundant in axons than dendrites and soma [48], nectin1 has been suggested to modulate the formation and remodeling of synapses [4]. The reduction or even loss of nectin1 in MEC neurons during critical developmental periods may therefore disrupt synaptic adhesion in a circuit- and thus subregion-specific manner, and impair structural plasticity and cognition consequently. Taken together, these findings suggest the nectin1-nectin3 system as molecular targets of early-life stress and modulators of the stress effects.

Distinct stress paradigms differentially regulate the expression patterns of nectins in several cortical structures. Stress exposure during the first postnatal week reduces hippocampal nectin1 and nectin3 levels immediately after stress ended [15], whereas prolonged exposure to stress for more than 3 weeks during development or/and adulthood reduces nectin3 but not nectin1 level in the hippocampus and the medial prefrontal cortex [13, 14, 17]. In addition, in adult mice with a mixed BALB/c;C57BL/6 background, early (P2–P9) but not later (P10–17) postnatal stress exposure increases *nectin1* mRNA level in the dorsal hippocampus [18]. In parahippocampal regions such as the perirhinal cortex, chronic adult stress reduces *nectin1* mRNA level [19]. Therefore, the timing and duration of stress exposure, genetic background, and the brain region examined are important factors that contribute to these discrepancies. Corticotropin-releasing hormone receptor 1 has been suggested to modulate the effects of early-life stress on nectin expression levels [15, 16, 49]. However, the molecular mechanisms for the interactions among stress, genetic background, gender, and age on nectin expression in various brain regions merit further investigations.

In summary, our data showed that stress exposure during a critical developmental period could suppress the expression of presynaptic nectin1 and postsynaptic nectin3 pairs in the entorhinal-hippocampal circuit that is important for mnemonic processes. Nectin1 and nectin3 are potential therapeutic targets for early-life stress-related psychiatric disorders.

## Supplementary information

Supplemental Information
