# Supplementary material for: Disrupted presynaptic nectin1-based neuronal adhesion in the entorhinal-hippocampal circuit contributes to early-life stress-induced memory deficits
Source: Transl Psychiatry. 2022 Apr 4;12:141. doi: 10.1038/s41398-022-01908-y (PMC8980071; doi:10.1038/s41398-022-01908-y)
Supplement: Supplementary file 1 — Supplemental Information [file 41398_2022_1908_MOESM1_ESM.doc]

**Supplemental Information for:**

**Disrupted presynaptic nectin1-based neuronal adhesion in the entorhinal-hippocampal circuit contributes to early-life stress-induced memory deficits**

Chen Wu, Qian Gong, Xue Xu, Ping Fang, Chi Wang, Jing-Ying Yu, Xing-Xing Wang, San-Hua Fang, Wen-Juan Chen, Hui-Fang Lou, Yu-Hui Liu, Liang Wang, Yi-Jun Liu, Wei Chen, and Xiao-Dong Wang

**Supplemental Information**

1. Supplemental Figures

2. Supplemental Table S1

**
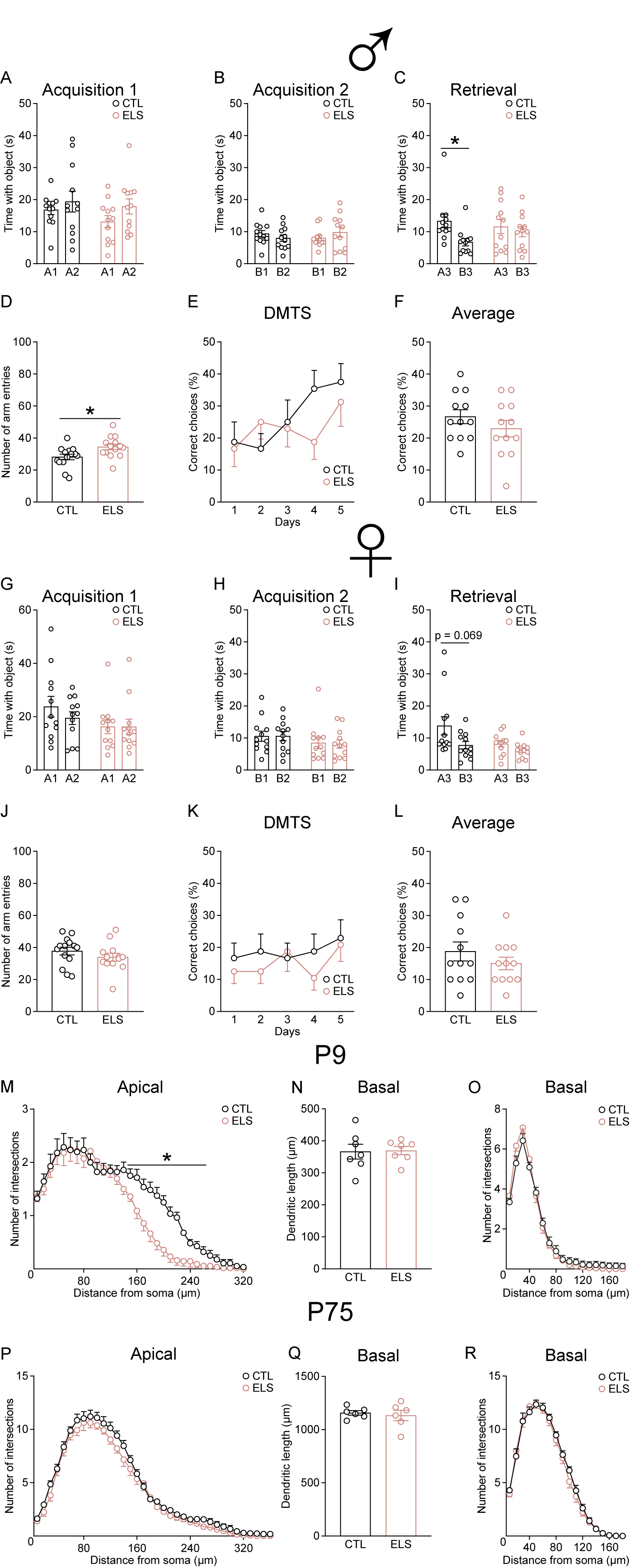
**

**Figure S1.** Effects of early-life stress on behavioral performance and the morphology of CA1 pyramidal neurons. (A) In the first acquisition phase of the temporal order task, adult male control (CTL) and early-life stressed (ELS) mice spent comparable time exploring the two identical cubes (A1 and A2). (B) In the second acquisition phase of the temporal order task, both groups of mice spent comparable time exploring the two identical cylinders (B1 and B2). (C) In the retrieval phase of the temporal order task, male CTL mice spent more time exploring the new copy of the cube (A3, “remote”) than the copy of the cylinder (B3, “recent”). In comparison, male ELS mice explored the two objects similarly. (D) In the Y-maze spontaneous alternation task, male ELS mice had more visits to all arms than CTL mice. (E and F) In the Y-maze delayed match-to-sample (DMTS) task, male CTL and ELS mice made comparable correct choices over the training sessions (E) and on average (F). n = 12 mice (A-C, E, and F) or 14 mice (D) per group. (G and H) In acquisition phases 1 (G) and 2 (H) of the temporal order task, adult female CTL and ELS mice explored the objects comparably. (I) In the retrieval phase of the temporal order task, female CTL mice spent relatively more time exploring the remote object than the recent one, while ELS mice failed to discriminate the objects. (J-L) In the Y-maze spontaneous alternation (J) and DMTS (K and L) tasks, no difference was found between groups. n = 12 mice per group for (G-I, K, and L). 14 CTL and 13 ELS mice were included in (J). (M-O) Sholl analysis of the apical and basal dendrites of CA1 pyramidal neurons in male pups on the 9^th^ postnatal day (abbreviated as P hereafter). The negative effects of early-life stress on apical dendrite branching were more prominent at 150-260 µm from the soma (M). By contrast, the total length (N) and the number of intersections at concentric circles (O) of basal dendrites were similar between groups. n = 7 mice per group. (P-R) Sholl analysis of the apical and basal dendrites of CA1 pyramidal neurons in adult male mice. The number of intersections at concentric circles of apical dendrites (P), the total length of basal dendrites (Q), and the number of intersections at concentric circles of basal dendrites (R) were not different between groups. n = 6 mice per group. *p < 0.05.

**
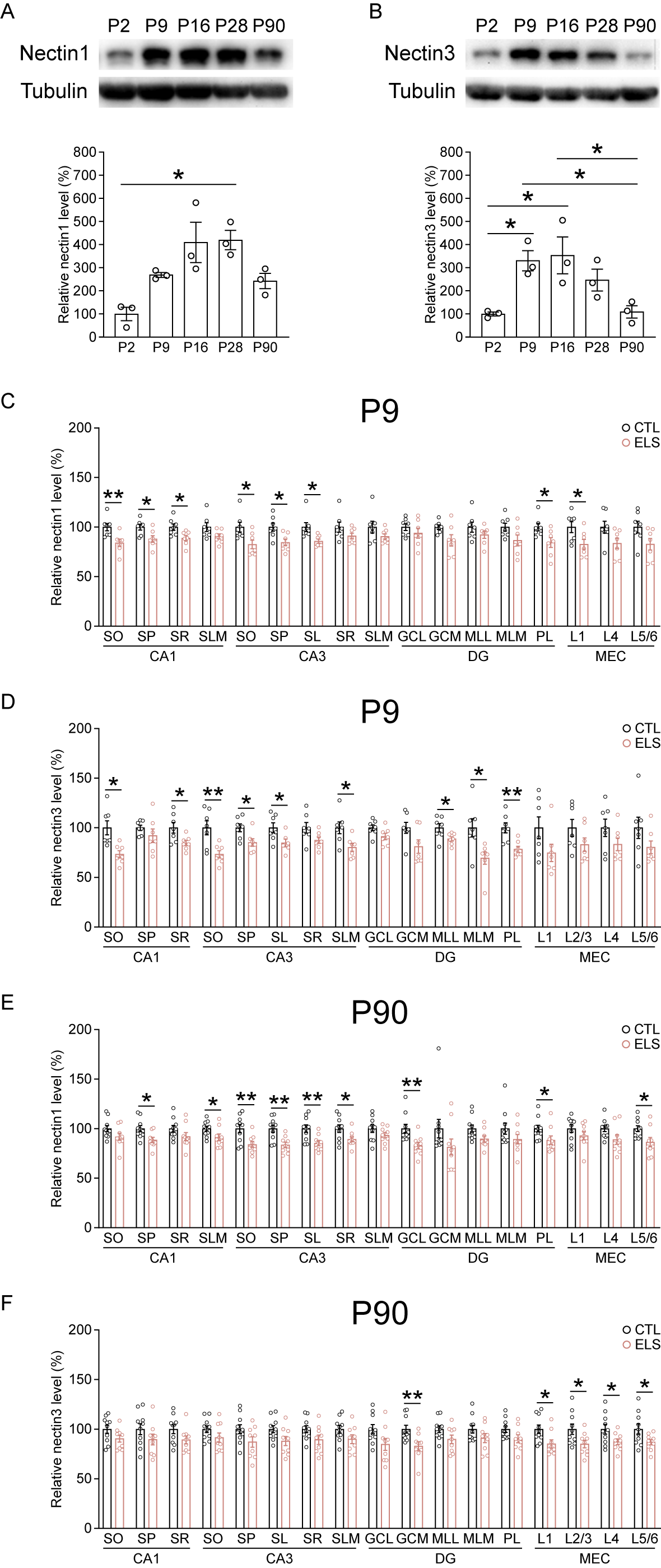
**

**Figure S2.** Immediate and lasting effects of early-life stress on nectin protein levels in medial entorhinal and hippocampal subregions. (A and B) Hippocampal nectin1 (A) and nectin3 (B) protein levels during development and young adulthood. Nectin1 level on P28 was higher than that on P2, while nectin3 levels on P9 and P16 were higher than those on P2 and P90. n = 3 mice per group (all test-naïve). (C and D) Quantification of nectin1 (C) and nectin3 (D) immunoreactivity in hippocampal and entorhinal subregions in control and stressed pups. n = 7 mice per group. (E and F) Quantification of nectin1 (E) and nectin3 (F) immunoreactivity in hippocampal and entorhinal subregions in adult mice with or without early-life adversities. n = 10 CTL and 9 ELS mice. SO, stratum oriens; SP, stratum pyramidale; SR, stratum radiatum; SLM, stratum lacunosum-moleculare; SL, stratum lucidum; DG, dentate gyrus; GCL, lateral part of granule cell layer; GCM, medial part of granule cell layer; MLL, lateral part of molecular layer; MLM, medial part of molecular layer; PL, polymorphic layer; MEC, medial entorhinal cortex; L, layer. *p < 0.05; **p < 0.01.

**
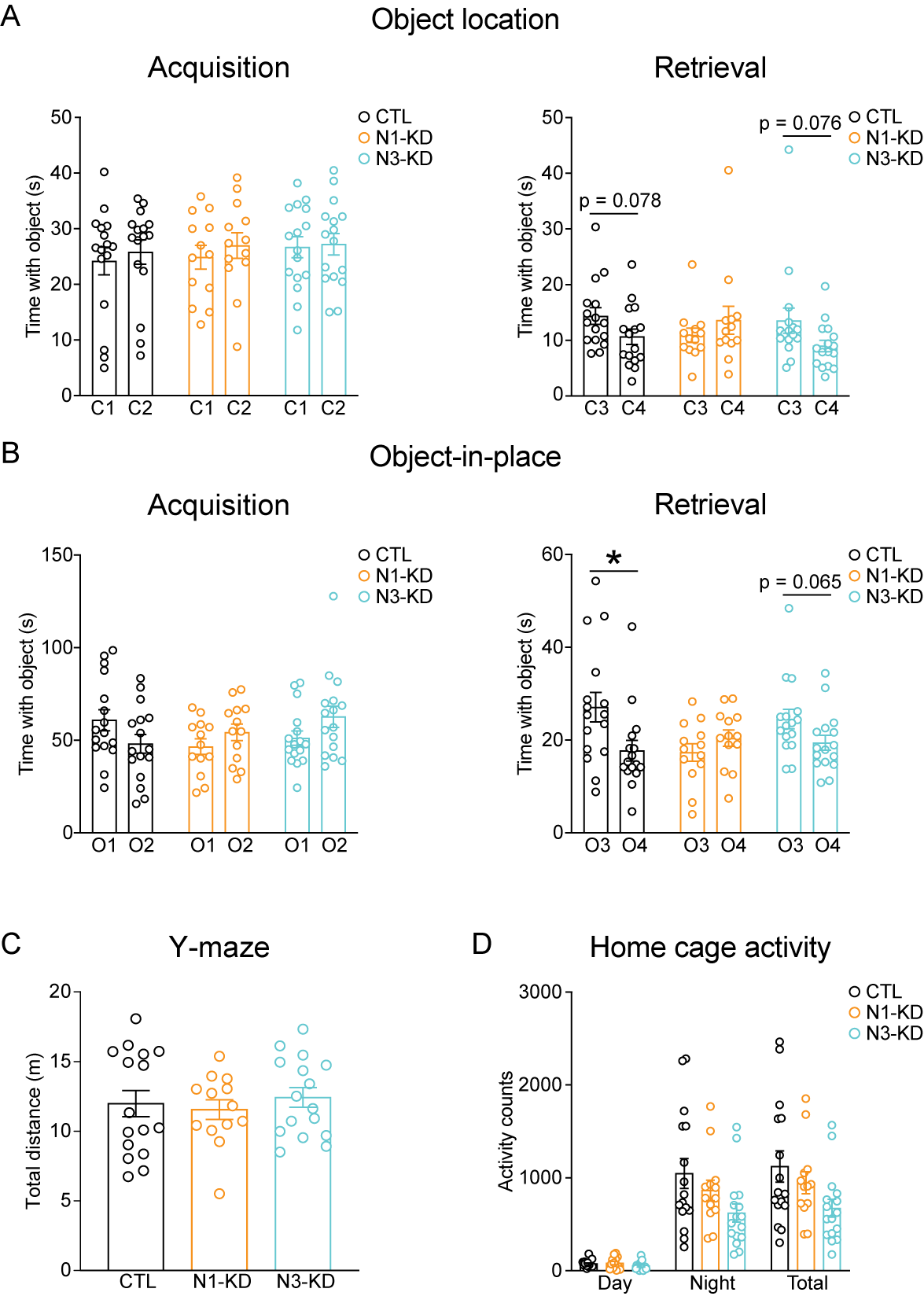
**

**Figure S3.** Effects of postnatal nectin1 or nectin3 knockdown in the MEC on behavioral performance. (A) In the acquisition phase of the object location task, all groups of mice spent comparable amount of time exploring the two circular cones (C1 and C2). In the retrieval phase, control and nectin3-knockdown (N3-KD) mice tended to spend more time exploring the relocated object (C3) than the non-displaced object (C4), while nectin1-knockdown (N1-KD) mice explored the two objects similarly. (B) In the acquisition phase of the object-in-place task, all groups of mice spent comparable amount of time exploring the four objects. In the retrieval phase, control mice spent more time exploring the relocated objects. N3-KD mice showed a trend to prefer the relocated objects, whereas N1-KD mice explored the two sets of objects similarly. O1 represents the two objects whose copies were exchanged in position in the retrieval phase (O3), while O2 represents the two objects whose copies remained in the same location in the retrieval phase (O4). (C) In the Y-maze spontaneous alternation task, postnatal knockdown of nectin1 nor nectin3 in the MEC did not affect general exploration level. (D) Knockdown of nectin1 nor nectin3 had no effect on home cage activity. 16 CTL, 13 N1-KD and 16 N3-KD mice were used. *p < 0.05.

**
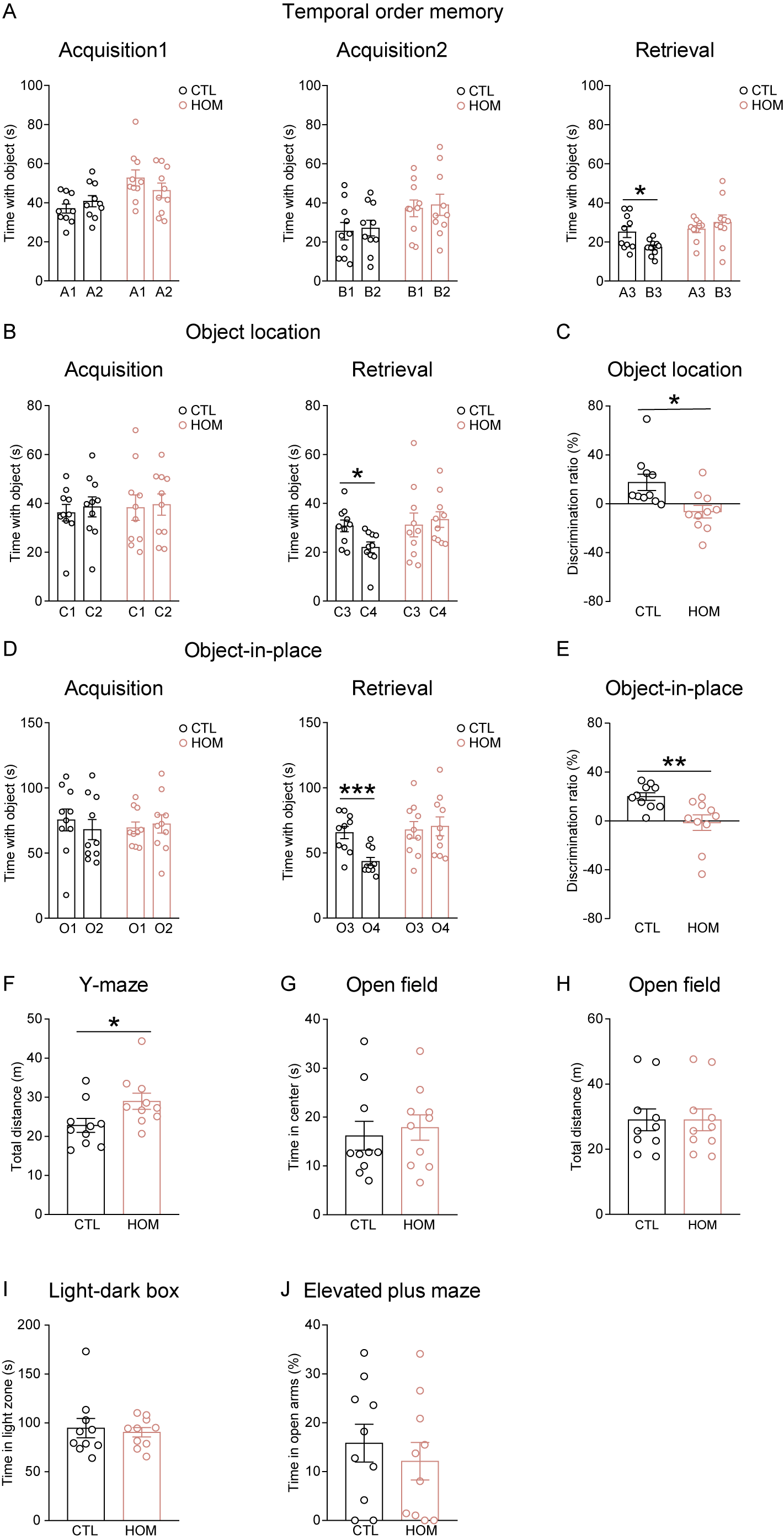
**

**Figure S4.** Effects of postnatal inactivation of nectin1 in MEC excitatory neurons on temporal order memory, spatial memory, and anxiety-related behavior. (A) AAV-Camk2α-Cre was delivered to the MEC of 2-day-old *nectin1^+/+^* (CTL) or *nectin1^loxP/loxP^* (HOM) pups. In the acquisition phases of the temporal order task, CTL and HOM mice explored the objects comparably. In the retrieval phase, CTL mice spent more time exploring the remote object than the recent one, while HOM mice failed to discriminate the objects. (B) In the acquisition phase of the object location task, both groups of mice spent comparable amount of time exploring the objects. In the retrieval phase, CTL but not HOM mice spent more time exploring the relocated object than the non-displaced one. (C) In the object location task, the discrimination ratio of HOM mice was significantly lower than that of CTL mice. (D) In the acquisition phase of the object-in-place task, both groups of mice spent comparable amount of time exploring the objects. In the retrieval phase, CTL but not HOM mice spent more time exploring the relocated objects than the stationary ones. (E) In the object-in-place task, the discrimination ratio of HOM mice was significantly lower than that of CTL mice. (F) In the Y-maze spontaneous alternation task, HOM mice traveled longer distances compared to CTL mice. (G and H) In the open field test, the amount of time in the center zone (G) and total distance traveled were not different between groups. (I) In the light-dark box test, CTL and HOM mice spent similar amount of time in the brightly illuminated chamber. (J) In the elevated plus maze test, CTL and HOM mice spent comparable time visiting the open arms. n = 10 mice per group. *p < 0.05; **p < 0.01; ***p < 0.001.


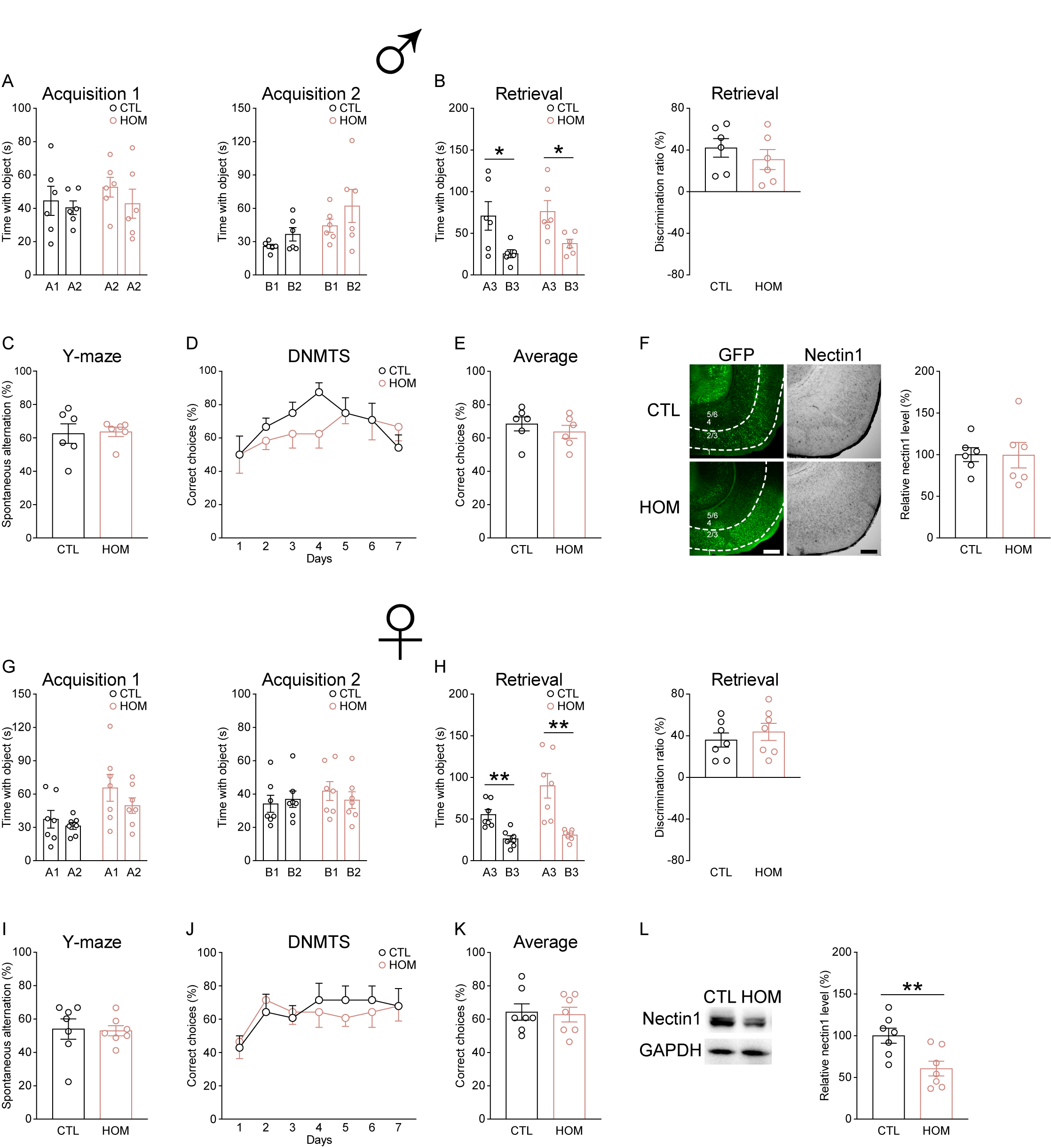


**Figure S5.** Cognitive effects of the control AAV injection to the MEC in male mice and conditional entorhinal nectin1 inactivation in female mice. (A) In acquisition phases of the temporal order task, adult male wild-type and HOM mice that both received AAV-Camk2α-GFP injection to the MEC on P2 explored the objects comparably. (B) In the retrieval phase, both groups spent more time exploring the remote object than the recent one, and had comparable object discrimination ratio. (C-E) In the Y-maze spontaneous alternation (C) and DNMTS (D and E) tasks, no difference was noticed between groups. (F) Microinjections of the control AAV to the MEC did not alter nectin1 level. Representative immunostaining images are shown. Scale bars are 200 µm. n = 6 mice per group. (G) In acquisition phases of the temporal order task, adult female wild-type and HOM mice that both received neonatal microinjections of AAV-Camk2α-Cre to the MEC explored the objects comparably. (H) In the retrieval phase, female HOM mice with entorhinal nectin1 deletion and control females spent significantly more time exploring the remote object than the recent one, and no between-group difference in discrimination ratio was found. (I-K) In the Y-maze spontaneous alternation (I) and DNMTS (J and K) tasks, both groups performed similarly. (L) Nectin1 protein level was significantly reduced in the MEC of female HOM mice. Representative immunoblots are shown. n = 7 mice per group. *p < 0.05; **p < 0.01.

**Table S1.** Statistical methods, results and sample size related to each figure.

| **Figure** | **Statistical method** | **Factors** | **Statistical results** | ***Post hoc* method** | ***Post hoc* results** | **Sample size** |
| --- | --- | --- | --- | --- | --- | --- |
| 2A | Two-tailed unpaired *t* test | Condition | *t*_22_ = 2.579, p = 0.017 |  |  | 12 mice per group |
| 2B | Two-tailed unpaired *t* test | Condition | *t*_26_ = 2.081, p = 0.047 |  |  | 14 mice per group |
| 2C | One-way repeated measures ANOVA | Condition | *F*_1,22_ = 10.586, p = 0.004 | Two-tailed unpaired *t* test | Day 1: *t*_22_ = 1.797, p = 0.086;  Day 2: *t*_22_ = 1.865, p = 0.076;  Day 3: *t*_22_ = 1.990, p = 0.059;  Day 4: *t*_22_ = 0.233, p = 0.818;  Day 5: *t*_22_ = 1.414, p = 0.171;  Day 6: *t*_22_ = 0.506, p = 0.618;  Day 7: *t*_22_ = 1.603, p = 0.123 | 12 mice per group |
| 2D | Two-tailed unpaired *t* test | Condition | *t*_22_ = 3.254, p = 0.004 |  |  | 12 mice per group |
| 2E | Two-tailed unpaired *t* test | Condition | *t*_22_ = 1.689, p = 0.105 |  |  | 12 mice per group |
| 2F | Two-tailed unpaired *t* test | Condition | *t*_25_ = 0.143, p = 0.888 |  |  | 14 CTL and 13 ELS mice |
| 2G | One-way repeated measures ANOVA | Condition | *F*_1,22_ = 2.839, p = 0.106 |  |  | 12 mice per group |
| 2H | Two-tailed unpaired *t* test | Condition | *t*_22_ = 1.685, p = 0.106 |  |  | 12 mice per group |
| 2J | Two-tailed unpaired *t* test | Condition | Apical: *t*_8.614_ = 3.128, p = 0.013; SR: *t*_12_ = 1.662, p = 0.122; SLM: *t*_12_ = 3.804, p = 0.003 |  |  | 7 mice per group |
| 2K | Two-tailed unpaired *t* test | Condition | *t*_12_ = 3.705, p = 0.003 |  |  | 7 mice per group |
| 2L | Two-tailed unpaired *t* test | Condition | Total: *t*_12_ = 1.805, p = 0.096; Thin: *t*_12_ = 0.464, p = 0.651; Mushroom: *t*_12_ = 4.467, p = 0.001;  Stubby: *t*_12_ = 2.134, p = 0.054; Filopodia: *t*_12_ = 0.060, p = 0.953 |  |  | 7 mice per group |
| 2N | Two-tailed unpaired *t* test | Condition | Apical: *t*_10_ = 2.469, p = 0.033; SR: *t*_10_ = 1.660, p = 0.128; SLM: *t*_10_ = 2.826, p = 0.018 |  |  | 6 mice per group |
| 2O | Two-tailed unpaired *t* test | Condition | *t*_10_ = 2.349, p = 0.041 |  |  | 6 mice per group |
| 2P | Two-tailed unpaired *t* test | Condition | Total: *t*_10_ = 0.004, p = 0.997; Thin: *t*_10_ = 0.022, p = 0.983; Mushroom: *t*_6.067_ = -0.812, p = 0.436;  Stubby: *t*_10_ = 0.478, p = 0.643 |  |  | 6 mice per group |
| 3A | Two-tailed unpaired *t* test | Condition | *t*_14_ = 2.774, p = 0.015 |  |  | 8 mice per group |
| 3B | Two-tailed unpaired *t* test | Condition | *t*_14_ = 3.835, p = 0.002 |  |  | 8 mice per group |
| 3C | Two-tailed unpaired *t* test | Condition | *t*_14_ = 3.133, p = 0.007 |  |  | 8 mice per group |
| 3D | Two-tailed unpaired *t* test | Condition | *t*_9.310_ = 2.808, p = 0.020 |  |  | 8 mice per group |
| 3E | One-way repeated measures ANOVA | Condition | Dorsal-ventral: *F*_1,12_ = 5.271, p = 0.040;  Relative nectin1 level (%): *t*_12_ = 2.290, p = 0.041 | Two-tailed unpaired *t* test | Level 1: *t*_12_ = 2.393, p = 0.034;  2: *t*_12_ = 1.981, p = 0.071;  3: *t*_12_ = 2.814, p = 0.016;  4: *t*_9.748_ = 1.201, p = 0.258;  5: *t*_12_ = 1.227, p = 0.206;  6: *t*_12_ = 1.080, p = 0.302;  7: *t*_12_ = 1.358, p = 0.199;  8: *t*_12_ = 0.607, p = 0.555 | 7 mice per group |
| 3F | One-way repeated measures ANOVA | Condition | Dorsal-ventral: *F*_1,12_ = 10.409, p = 0.007;  Relative nectin3 level (%): *t*_12_ = 3.265, p = 0.013 | Two-tailed unpaired *t* test | Level 1: *t*_12_ = 1.163, p = 0.267;  2: *t*_11.693_ = 2.332, p = 0.038;  3: *t*_12_ = 1.574, p = 0.142;  4: *t*_7.066_ = 0.855, p = 0.421;  5: *t*_12_ = 0.167, p = 0.870;  6: *t*_12_ = 2.889, p = 0.014;  7: *t*_12_ = 1.564, p = 0.144;  8: *t*_12_ = 1.928, p = 0.078 | 7 mice per group |
| 3G | One-way repeated measures ANOVA | Condition | Dorsal-ventral: *F*_1,17_ = 5.212, p = 0.036;  Relative nectin1 level (%): *t*_17_ = 2.211, p = 0.041 | Two-tailed unpaired *t* test | Level 1: *t*_17_ = 0.620, p = 0.543;  2: *t*_17_ = 0.513, p = 0.615;  3: *t*_17_ = 1.472, p = 0.159;  4: *t*_17_ = 1.420, p = 0.174;  5: *t*_17_ = 2.189, p = 0.043;  6: *t*_17_ = 1.136, p = 0.272;  7: *t*_17_ = 1.368, p = 0.189;  8: *t*_17_ = 3.705, p = 0.002 | 10 CTL and 9 ELS mice |
| 3H | One-way repeated measures ANOVA | Condition | Dorsal-ventral: *F*_1,17_ = 5.125, p = 0.037;  Relative nectin3 level (%): *t*_17_ = 2.264, p = 0.037 | Two-tailed unpaired *t* test | Level 1: *t*_17_ = 0.368, p = 0.717;  2: *t*_17_ = 0.166, p = 0.870;  3: *t*_17_ = 0.110, p = 0.914;  4: *t*_17_ = 0.638, p = 0.532;  5: *t*_17_ = 2.910, p = 0.010;  6: *t*_17_ = 4.506, p < 0.001;  7: *t*_17_ = 1.676, p = 0.112;  8: *t*_17_ = 3.290, p = 0.004 | 10 CTL and 9 ELS mice |
| 4C | One-way ANOVA | Condition | *F*_2,42_ = 10.548, p < 0.001 | Dunnett’s test | N1-KD versus CTL, p = 0.002; N1-KD versus N3-KD, p < 0.001;  CTL versus N3-KD, p = 0.999 | 16 CTL, 13 N1-KD and 16 N3-KD mice |
| 4D | One-way ANOVA | Condition | *F*_2,42_ = 8.431, p < 0.001 | Tukey’s test | N1-KD versus CTL, p = 0.002; N1-KD versus N3-KD, p = 0.017;  CTL versus N3-KD, p = 0.441 | 16 CTL, 13 N1-KD and 16 N3-KD mice |
| 4E | One-way ANOVA | Condition | *F*_2,41_ = 1.213, p = 0.308 |  |  | 15 CTL, 13 N1-KD and 16 N3-KD mice |
| 4F | Two-tailed unpaired *t* test | Condition | *t*_10_ = 4.694, p < 0.001 |  |  | 6 mice per group |
| 4G | Two-tailed unpaired *t* test | Condition | *t*_10_ = 0.068, p = 0.947 |  |  | 6 mice per group |
| 4H | Two-tailed unpaired *t* test | Condition | *t*_10_ = 8.010, p < 0.001 |  |  | 6 mice per group |
| 4I | Two-tailed unpaired *t* test | Condition | *t*_10_ = 1.957, p = 0.079 |  |  | 6 mice per group |
| 5C | One-way ANOVA | Condition | *F*_2,9_ = 12.319, p = 0.003 | Tukey’s test | CTL versus HOM: p = 0.002;  CTL versus HET: p = 0.150;  HOM versus HET: p = 0.096 | 4 CTL, 3 HET and 5 HOM mice |
| 5D | One-way ANOVA | Condition | *F*_2,9_ = 0.095, p = 0.910 |  |  | 4 CTL, 3 HET and 5 HOM mice |
| 5F | Two-tailed unpaired *t* test | Condition | *t*_10_ = 2.881, p = 0.016 |  |  | 6 mice per group |
| 5G | Two-tailed unpaired *t* test | Condition | *t*_18_ = 3.435, p = 0.003 |  |  | 10 mice per group |
| 5H | Two-tailed unpaired *t* test | Condition | *t*_18_ = 2.388*,* p = 0.028 |  |  | 10 mice per group |
| 5I | One-way repeated measures ANOVA | Condition | *F*_1,18_ = 5.516, p = 0.030 | Two-tailed unpaired *t* test | Day 1: *t*_18_ = 0, p = 1.000;  Day 2: *t*_18_ = -3.539, p = 0.002;  Day 3: *t*_18_ = 0, p = 1.000;  Day 4: *t*_18_ = 0.424, p = 0.676;  Day 5: *t*_18_ = 1.909, p = 0.072;  Day 6: *t*_18_ = 0.920, p = 0.370;  Day 7: *t*_13.235_ = 3.806, p = 0.002 | 10 mice per group |
| 5J | Two-tailed unpaired *t* test | Condition | *t*_18_ = 2.954, p = 0.008 |  |  | 10 mice per group |
| 5L | Two-tailed unpaired *t* test | Condition | Apical: *t*_10_ = 1.100, p = 0.297; SR: *t*_10_ = -1.221, p = 0.250; SLM: *t*_10_ = 5.669, p < 0.001 |  |  | 6 mice per group |
| 5M | Two-tailed unpaired *t* test | Condition | *t*_10_ = 6.937, p < 0.001 |  |  | 6 mice per group |
| 5N | One-way repeated measures ANOVA | Condition | *F*_1,10_ = 2.006, p = 0.187 | Two-tailed unpaired *t* test | Distance from soma 300: *t*_10_ = 2.453, p = 0.034;  310: *t*_10_ = 3.081, p = 0.012;  320: *t*_10_ = 3.625, p = 0.005;  330: *t*_10_ = 4.195, p = 0.002;  340: *t*_10_ = 3.297, p = 0.008;  350: *t*_10_ = 2.257, p = 0.048;  360: *t*_10_ = 2.329, p = 0.042 | 6 mice per group |
| S1A | Two-tailed unpaired *t* test | Condition | CTL: *t*_15.326_ = -0.728, p = 0.474; ELS: *t*_22_ = -1.566, p = 0.132 |  |  | 12 mice per group |
| S1B | Two-tailed unpaired *t* test | Condition | CTL: *t*_22_ = 0.963, p = 0.346; ELS: *t*_17.025_ = -1.034, p = 0.316 |  |  | 12 mice per group |
| S1C | Two-tailed unpaired *t* test | Condition | CTL: *t*_22_ = 2.794, p = 0.011; ELS: *t*_22_ = 0.566, p = 0.577 |  |  | 12 mice per group |
| S1D | Two-tailed unpaired *t* test | Condition | *t*_26_ = -2.569, p = 0.016 |  |  | 14 mice per group |
| S1E | One-way repeated measures ANOVA | Condition | *F*_1,22_ = 1.246, p = 0.276 |  |  | 12 mice per group |
| S1F | Two-tailed unpaired *t* test | Condition | *t*_22_ = 1.116, p = 0.276 |  |  | 12 mice per group |
| S1G | Two-tailed unpaired *t* test | Condition | CTL: *t*_22_ = 0.945, p = 0.355; ELS: *t*_22_ = -0.002, p = 0.998 |  |  | 12 mice per group |
| S1H | Two-tailed unpaired *t* test | Condition | CTL: *t*_22_ = -0.012, p = 0.991; ELS: *t*_22_ = 0.168, p = 0.868 |  |  | 12 mice per group |
| S1I | Two-tailed unpaired *t* test | Condition | CTL: *t*_14.545_ = 1.962, p = 0.069; ELS: *t*_22_ = 1.679, p = 0.107 |  |  | 12 mice per group |
| S1J | Two-tailed unpaired *t* test | Condition | *t*_25_ = 1.103, p = 0.280 |  |  | 14 CTL and 13 ELS mice |
| S1K | One-way repeated measures ANOVA | Condition | *F*_1,22_ = 1.121, p = 0.301 |  |  | 12 mice per group |
| S1L | Two-tailed unpaired *t* test | Condition | *t*_22_ = 1.059, p = 0.301 |  |  | 12 mice per group |
| S1M | One-way repeated measures ANOVA | Condition | *F*_1,12_ = 10.237, p = 0.008 | Two-tailed unpaired *t* test | Distance from soma A150: *t*_12_ = 2.538, p = 0.026;  A160: *t*_12_ = 2.902, p = 0.013;  A170: *t*_12_ = 3.270, p = 0.007;  A180: *t*_12_ = 3.898, p = 0.002;  A190: *t*_12_ = 4.514, p = 0.001;  A200: *t*_12_ = 5.354, p < 0.001;  A210: *t*_12_ = 4.822, p < 0.001;  A220: *t*_12_ = 5.943, p < 0.001;  A230: *t*_12_ = 5.370, p < 0.001;  A240: *t*_12_ = 2.679, p = 0.20;  A250: *t*_12_ = 3.241, p = 0.007;  A260: *t*_12_ = 2.645, p = 0.021 | 7 mice per group |
| S1N | Two-tailed unpaired *t* test | Condition | *t*_12_ = -0.117, p = 0.909 |  |  | 7 mice per group |
| S1O | One-way repeated measures ANOVA | Condition | *F*_1,12_ = 0.371, p = 0.554 |  |  | 7 mice per group |
| S1P | One-way repeated measures ANOVA | Condition | *F*_1,10_ = 4.149, p = 0.069 |  |  | 6 mice per group |
| S1Q | Two-tailed unpaired *t* test | Condition | *t*_10_ = 0.458, p = 0.657 |  |  | 6 mice per group |
| S1R | One-way repeated measures ANOVA | Condition | *F*_1,10_ = 0.242, p = 0.633 |  |  | 6 mice per group |
| S2A | One-way ANOVA | Condition | *F*_4,10_ = 7.646, p = 0.004 | Dunnett’s test | P28 versus P2, p = 0.026 | 3 mice per group |
| S2B | One-way ANOVA | Condition | *F*_4,10_ = 6.358, p = 0.008 | Tukey’s test | P9 versus P2, p = 0.04;  P16 versus P2, p = 0.023;  P9 versus P90, p = 0.049;  P16 versus P90, p = 0.029 | 3 mice per group |
| S2C | Two-tailed unpaired *t* test | Condition | **CA1**  SO: *t*_12_ = 3.070, p = 0.0097;  SP: *t*_12_ = 2.707, p = 0.019;  SR: *t*_12_ = 2.629, p = 0.022;  **CA3**  SO: *t*_12_ = 2.667, p = 0.021;  SP: *t*_12_ = 2.897, p = 0.013;  SL: *t*_12_ = 2.822, p = 0.015;  **DG**  PL: *t*_12_ = 2.466, p = 0.03;  **MEC**  L1: *t*_12_ = 2.345, p = 0.037 |  |  | 7 mice per group |
| S2D | Two-tailed unpaired *t* test | Condition | **CA1**  SO: *t*_12_ = 2.535, p = 0.026;  SR: *t*_12_ = 2.550, p = 0.025;  **CA3**  SO: *t*_12_ = 3.331, p = 0.006;  SP: *t*_12_ = 2.689, p = 0.02;  SL: *t*_12_ = 2.631, p = 0.022;  SLM: *t*_12_ = 2.823, p = 0.015;  **DG**  MLL: *t*_12_ = 2.341, p = 0.037;  MLM: *t*_12_ = 2.746, p = 0.018;  PL: *t*_12_ = 3.633, p = 0.003 |  |  | 7 mice per group |
| S2E | Two-tailed unpaired *t* test | Condition | **CA1**  SP: *t*_17_ = 2.786, p = 0.013;  SLM: *t*_17_ = 2.155, p = 0.046;  **CA3**  SO: *t*_17_ = 3.02, p = 0.008;  SP: *t*_17_ = 3.585, p = 0.002;  SL: *t*_17_ = 3.102, p = 0.002;  SR: *t*_17_ = 2.305, p = 0.034;  **DG**  GCL: *t*_17_ = 3.065, p = 0.007;  PL: *t*_17_ = 2.161, p = 0.045;  **MEC**  L5/6: *t*_17_ = 2.515, p = 0.022 |  |  | 10 CTL and 9 ELS mice |
| S2F | Two-tailed unpaired *t* test | Condition | **DG**  GCM: *t*_17_ = 2.191, p = 0.0096;  **MEC**  L1: *t*_17_ = 2.514, p = 0.022;  L2/3: *t*_17_ = 2.283, p = 0.036;  L4: *t*_17_ = 2.137, p = 0.047;  L5/6: *t*_17_ = 2.125, p = 0.049 |  |  | 10 CTL and 9 ELS mice |
| S3A | Two-tailed unpaired *t* test | Condition | **Acquisition**  CTL: *t*_30_ = 0.487, p = 0.630;  N1-KD: *t*_24_ = 0.673, p = 0.508;  N3-KD: *t*_30_ = 0.197, p = 0.845;  **Retrieval**  CTL: *t*_30_ = -1.824, p = 0.078;  N1-KD: *t*_24_ = 0.960, p = 0.347;  N3-KD: *t*_30_ = -1.836, p = 0.076 |  |  | 16 CTL, 13 N1-KD and 16 N3-KD mice |
| S3B | Two-tailed unpaired *t* test | Condition | **Acquisition**  CTL: *t*_30_ = -1.675, p = 0.104;  N1-KD: *t*_24_ = 1.259, p = 0.220;  N3-KD: *t*_30_ = 1.656, p = 0.108;  **Retrieval**  CTL: *t*_30_ = -2.406, p = 0.023;  N1-KD: *t*_24_ = 1.190, p = 0.246;  N3-KD: *t*_30_ = -1.921, p = 0.065 |  |  | 16 CTL, 13 N1-KD and 16 N3-KD mice |
| S3C | One-way ANOVA | Condition | *F*_2,41_ = 0.282, p = 0.756 |  |  | 16 CTL, 13 N1-KD and 16 N3-KD mice |
| S3D | One-way ANOVA | Condition | Day: *F*_2,42_ = 1.693, p = 0.196;  Night: *F*_2,42_ = 2.940, p = 0.064; Total: *F*_2,42_ = 3.084, p = 0.056 |  |  | 16 CTL, 13 N1-KD and 16 N3-KD mice |
| S4A | Two-tailed unpaired *t* test | Condition | **Acquisition 1**  CTL: *t*_18_ = -1.022, p = 0.320; HOM: *t*_18_ = 1.151, p = 0.265; **Acquisition 2**  CTL: *t*_18_ = -0.258, p = 0.799; HOM: *t*_18_ = -0.260, p = 0.798;  **Retrieval**  CTL: *t*_12.422_ = 2.566, p = 0.024; HOM: *t*_18_ = -0.843, p = 0.410 |  |  | 10 mice per group |
| S4B | Two-tailed unpaired *t* test | Condition | **Acquisition**  CTL: *t*_18_ = -0.457, p = 0.653; HOM: *t*_18_ = -0.182, p = 0.858;  **Retrieval**  CTL: *t*_18_ = -2.674, p = 0.015; HOM: *t*_18_ = 0.380, p = 0.708 |  |  | 10 mice per group |
| S4C | Two-tailed unpaired *t* test | Condition | *t*_18_ = 2.839, p = 0.011 |  |  | 10 mice per group |
| S4D | Two-tailed unpaired *t* test | Condition | **Acquisition**  CTL: *t*_18_ = 0.653, p = 0.522; HOM: *t*_18_ = -0.364, p = 0.720;  **Retrieval**  CTL: *t*_18_ = -3.943, p < 0.001;  HOM: *t*_18_ = 0.285, p = 0.779 |  |  | 10 mice per group |
| S4E | Two-tailed unpaired *t* test | Condition | *t*_18_ = 3.027, p = 0.007 |  |  | 10 mice per group |
| S4F | Two-tailed unpaired *t* test | Condition | *t*_18_ = -2.263*,* p = 0.036 |  |  | 10 mice per group |
| S4G | Two-tailed unpaired *t* test | Condition | *t*_18_ = -0.431, p = 0.672 |  |  | 10 mice per group |
| S4H | Two-tailed unpaired *t* test | Condition | *t*_18_ = -0.810, p = 0.428 |  |  | 10 mice per group |
| S4I | Two-tailed unpaired *t* test | Condition | *t*_18_ = 0.386, p = 0.704 |  |  | 10 mice per group |
| S4J | Two-tailed unpaired *t* test | Condition | *t*_18_ = 0.679, p = 0.506 |  |  | 10 mice per group |
| S5A | Two-tailed unpaired *t* test | Condition | **Acquisition 1**  CTL: *t*_10_ = 0.430, p = 0.677; HOM: *t*_10_ = 0.938, p = 0.370; **Acquisition 2**  CTL: *t*_5.911_ = -1.727, p = 0.136; HOM: *t*_6.557_ = -1.110, p = 0.306 |  |  | 6 mice per group |
| S5B | Two-tailed unpaired *t* test | Condition | **Time with object**  CTL: *t*_5.765_ = 2.551, p = 0.045; HOM: *t*_6.519_ = 2.792, p = 0.031;  **Discrimination ratio**  *t*_10_ = 0.850, p = 0.415 |  |  | 6 mice per group |
| S5C | Two-tailed unpaired *t* test | Condition | *t*_10_ = -0.163, p = 0.875 |  |  | 6 mice per group |
| S5D | One-way repeated measures ANOVA | Condition | *F*_1,10_ = 0.705, p = 0.421 |  |  | 6 mice per group |
| S5E | Two-tailed unpaired *t* test | Condition | *t*_10_ = 0.84, p = 0.421 |  |  | 6 mice per group |
| S5F | Two-tailed unpaired *t* test | Condition | *t*_10_ = 0.034, p = 0.973 |  |  | 6 mice per group |
| S5G | Two-tailed unpaired *t* test | Condition | **Acquisition 1**  CTL: *t*_12_ = 0.701, p = 0.496; HOM: *t*_12_ = 1.148, p = 0.273; **Acquisition 2**  CTL: *t*_12_ = -0.388, p = 0.705; HOM: *t*_12_ = 0.723, p = 0.483 |  |  | 7 mice per group |
| S5H | Two-tailed unpaired *t* test | Condition | **Time with object**  CTL: *t*_12_ = 4.150, p = 0.0013; HOM: *t*_6.322_ = 3.948, p = 0.007;  **Discrimination ratio**  *t*_12_ = -0.733, p = 0.478 |  |  | 7 mice per group |
| S5I | Two-tailed unpaired *t* test | Condition | *t*_12_ = 0.129, p = 0.899 |  |  | 7 mice per group |
| S5J | One-way repeated measures ANOVA | Condition | *F*_1,12_ = 0.054, p = 0.821 |  |  | 7 mice per group |
| S5K | Two-tailed unpaired *t* test | Condition | *t*_12_ = 0.232, p = 0.821 |  |  | 7 mice per group |
| S5L | Two-tailed unpaired *t* test | Condition | *t*_12_ = 3.130, p = 0.009 |  |  | 7 mice per group |
